# Age-dependent changes on fractalkine forms and their contribution to neurodegenerative diseases

**DOI:** 10.3389/fnmol.2023.1249320

**Published:** 2023-09-25

**Authors:** Jaime Eugenín, Laura Eugenín-von Bernhardi, Rommy von Bernhardi

**Affiliations:** ^1^Facultad de Química y Biología, Departamento de Biología, Universidad de Santiago de Chile, USACH, Santiago, Chile; ^2^Complejo Asistencial Hospital Dr. Sótero del Río, Santiago, Chile; ^3^Facultad de Ciencias para el Cuidado de la Salud, Universidad San Sebastián, Santiago, Chile

**Keywords:** aging, Alzheimer’s disease, CX3CL1, CX3CR1, metalloproteases, neurodegenerative disease, neuroinflammation, Parkinson’s disease

## Abstract

The chemokine fractalkine (FKN, CX_3_CL1), a member of the CX_3_C subfamily, contributes to neuron–glia interaction and the regulation of microglial cell activation. Fractalkine is expressed by neurons as a membrane-bound protein (mCX_3_CL1) that can be cleaved by extracellular proteases generating several sCX_3_CL1 forms. sCX_3_CL1, containing the chemokine domain, and mCX_3_CL1 have high affinity by their unique receptor (CX_3_CR1) which, physiologically, is only found in microglia, a resident immune cell of the CNS. The activation of CX_3_CR1contributes to survival and maturation of the neural network during development, glutamatergic synaptic transmission, synaptic plasticity, cognition, neuropathic pain, and inflammatory regulation in the adult brain. Indeed, the various CX_3_CL1 forms appear in some cases to serve an anti-inflammatory role of microglia, whereas in others, they have a pro-inflammatory role, aggravating neurological disorders. In the last decade, evidence points to the fact that sCX_3_CL1 and mCX_3_CL1 exhibit selective and differential effects on their targets. Thus, the balance in their level and activity will impact on neuron–microglia interaction. This review is focused on the description of factors determining the emergence of distinct fractalkine forms, their age-dependent changes, and how they contribute to neuroinflammation and neurodegenerative diseases. Changes in the balance among various fractalkine forms may be one of the mechanisms on which converge aging, chronic CNS inflammation, and neurodegeneration.

## 1. Introduction

The aging of the population is a major challenge in public health because aging is the main risk factor for many chronic diseases (Liguori et al., 2018), including neurodegenerative diseases (Hou et al., 2019). Additionally, demographic projections show that the elderly *population* is growing at an unprecedented rate (Partridge et al., 2018). A relevant feature of physiological aging is a *low*-*grade* chronic systemic *inflammation. This has been called “inflamm-aging,”* a condition that increases, *per se,* the mortality and morbidity among older adults (Franceschi et al., 2000).

Several age-related changes in neural function are associated with glial dysregulation (von Bernhardi, 2007). Age-related changes in glial cells functions can exert beneficial or detrimental influence onto neuronal circuits and, therefore, modify the course of the aging process (Lynch et al., 2010). However, the role of glial cells on the function/dysfunction of the aged central nervous system (CNS) is still poorly understood. For instance, glia may contribute to the age-related chronic inflammatory state of the nervous system (Franceschi et al., 2000, 2007; Franceschi, 2007). That is, glia can contribute to create a “pathological environment” providing deleterious factors that acting synergistically, can favor neuronal death and neurodegeneration (von Bernhardi and Eugenin, 2012). These changes can be crucial in cognitive impairment, failure of vital functions, and they can be part of the pathogenesis of highly prevalent CNS pathologies like neurodegenerative and neuropsychiatric diseases (Wu et al., 2015; Hong et al., 2016).

Therefore, it is not a surprise that changes or impairment of neuron-glial signaling will impact neuronal and glial properties at multiple levels, including synapse integrity and plasticity, network excitability, up to animal behavior (Wu et al., 2015). Here we will discuss age-dependent changes in fractalkine (FKN, CX_3_CL1) signaling, on the balance among soluble (sCX_3_CL1) vs. membrane-bound CX_3_CL1 (mCX_3_CL1) forms, and how changes in this balance contributes to switching microglia from an anti-inflammatory to a pro-inflammatory status, a key mechanism likely involved in chronic CNS inflammation and neurodegenerative diseases.

### 1.1. Aging

Aging affects cells, organs, and functions in various ways, particularly in the CNS (Ramirez et al., 2008; von Bernhardi et al., 2019). Aging is characterized by a low-grade inflammatory condition and the progressive deterioration of various physiological functions of the organism, being recognized as the most robust risk factor for neurodegenerative diseases like Alzheimer’s disease (AD) (Guerreiro and Bras, 2015). The onset of the changes characteristic of diseases associated with aging is unclear (Lynch and Smith, 2005). Often, neurodegenerative diseases develop progressively, for many years before their clinical manifestations. For instance, in a patient diagnosed with AD at age 75, pathophysiological changes started when the person was 50–55 years old. Aged individuals have also changes on the immune system, known as immune senescence (Jones et al., 2010), characterized by decreased adaptive immunity, making the subject more susceptible to infections and tumors, and exacerbated innate immune function (von Bernhardi et al., 2019) favoring chronic neuroinflammation, which appears to be also associated with the appearance of neurodegenerative pathologies (Block et al., 2007; von Bernhardi, 2007; Gao and Hong, 2008).

### 1.2. Age-dependent changes of brain cells

A functional hallmark of brain aging (Gonzales et al., 2022), often occurring in the absence of neurological disease, is cognitive impairment, especially some memory and learning deficits. Although cell loss is minimal in most brain regions, in regions such as the hippocampus it may reach from 10 to 60% (Coleman and Flood, 1987). Functional decline can also be the result of exacerbated synapse pruning depending on the dysregulation of astrocytes and microglia (Paolicelli et al., 2011; Faust et al., 2021).

#### 1.2.1. Neurons

Aging in humans is associated with a reduction in total brain volume (Hedman et al., 2012), being the reduction of gray matter volume more pronounced than that of white matter (Ge et al., 2002). The annual rate of volume loss is about 0.2% after 35 years of age, which increases to 0.5% at age of 60, becoming over 0.5% after 60 (Hedman et al., 2012). Age-dependent cortical thinning, which is observed across most brain regions, is more accentuated in the frontal lobe (Raz et al., 1997; Salat et al., 2004). Although decline in hippocampus CA1 region, hilus, and subiculum, volumes appear to be a consequence of neuronal loss, in other regions, like the frontal cortex, the number of neurons are relatively preserved (August et al., 2022).

The brain undergoes a myriad of changes during aging that can lead to an atrophic aged brain. Neurons show dendrite retraction, especially in the prefrontal cortex and hippocampus. With increasing age, dendrites shrink, their branching become less complex, and lose spines (Dickstein et al., 2013), affecting inter-neuronal connections. There are also age-dependent changes in the synthesis of neurotransmitters. For instance, older people brains synthesize less dopamine, and have fewer receptors (Yin et al., 2014). Older adults with mild cognitive impairment appear to have less serotonin (Yin et al., 2014), which could potentiate memory loss. Functional effects are especially relevant for processes that require a high degree of synaptic plasticity. The number of synapses is reduced, which can affect learning and memory, facilitating cognitive decline. Although synaptic changes are selective and mild, they appear to affect cognitive decline beyond their changes in structure and neurotransmitters. The myelin wrapping of axons thins out (Bender et al., 2016), reducing the speed of propagation of action potentials, and impacting neuronal communication. Neurogenesis also declines with age (Li Puma et al., 2020); interestingly, in humans and rodents, physiological strategies that boost neurogenesis, such as regular exercise, can improve cognitive function (Ma et al., 2017). Studies on adult hippocampal neurogenesis in AD mouse models show a severe decline of neurogenesis before cognitive impairment (Li Puma et al., 2020).

Neurons are post-mitotic cells, arrested in the G0 phase of the cell cycle. Therefore, recognition of neuronal senescence cannot be performed on basis of proliferation arrest. A growing body of evidence indicates that cell senescence-like changes in neurons can be observed in aging brains. These characteristics involve shortening of telomeres (Ain et al., 2018), senescence-associated secretory phenotype (SASP), altered morphology and proteostasis, propensity to undergo apoptosis, autophagy impairment, accumulation of lipid droplets, increased activity of senescence-associated-b-galactosidase (SA-b-gal), and epigenetic alterations, including DNA methylation, chromatin remodeling, and histone post-translational modifications that affect gene expression (Di Micco et al., 2021; Sikora et al., 2021; Sahu et al., 2022).

#### 1.2.2. Oligodendrocytes

Oligodendrocytes are the myelinating cells of the CNS, generated from oligodendrocyte progenitor cells (OPCs), also known as NG2 cells, because they express the proteoglycan, NG2 (Bradl and Lassmann, 2010; Kuhn et al., 2019). The density of NG2(+) cells in white matter is 1.5-fold higher than that in gray matter (Dawson et al., 2003) and their morphology in the mature CNS vary from region to region (Hughes et al., 2013). NG2 cells maintain their migrating, proliferating, and differentiating capacities throughout life to generate oligodendrocytes and regenerate myelin (Young et al., 2013). In the white matter of the adult mouse brain, NG2 cells are continuously generating mature, myelinating oligodendrocytes, whereas in the gray matter, they mostly generate postmitotic NG2 cells (Dimou et al., 2008). In adult mice, NG2 cells proliferate and differentiate in response to several stimuli such as voluntary physical activity in a brain region-dependent manner. Furthermore, the physical activity-associated cognitive improvement is abolished when NG2 cells differentiation is prevented (Eugenin von Bernhardi and Dimou, 2022).

In addition to increasing the speed of propagation of the action potential, oligodendrocytes provide lactate and pyruvate as metabolic support for neurons (Lee et al., 2012; Franklin and Ffrench-Constant, 2017; Rawji et al., 2023). These functions are lost with the loss of oligodendrocytes observed in demyelinating diseases like multiple sclerosis (MS) resulting in lack of trophic support, leading to slowing of axonal conduction of action potentials, and irreversible degeneration of demyelinated axons (Franklin and Ffrench-Constant, 2017).

The decline in white matter volume with normal aging (Taubert et al., 2020), is thought to contribute to age-dependent cognitive impairment. In aging rodent and nonhuman primates, electron microscopy revealed myelin disintegration and decompaction with accumulation of electron-dense cytoplasm within some sheaths, suggestive of myelin breakdown (Peters and Sethares, 2003; Pannese, 2011). Some myelinated nerve fibers in white matter degenerate and disappear, whereas others show degeneration restricted to myelin sheaths, but axons remain intact (Peters, 2009). Remyelination of these axons results in thinner myelin sheaths with shorter internodes than that observed in young fibers (Peters and Sethares, 2003). This remyelination depends on oligodendrogenesis in the spinal cord, where half of newly generated cells expressed NG2 (Lasiene et al., 2009). Multiphoton live imaging of the upper layers of the cortex in mice has confirmed the occurrence of degeneration and decrease of internodes with advancing age (Hill et al., 2018).

Single-cell RNA sequencing revealed age-associated transcriptomic changes in mice brains. Aging oligodendrocytes show downregulation of genes encoding myelin proteins (*Mog*, *Plp*, and *Cnp*) and cholesterol synthesis pathway (*Hmgcs1*). By contrast, aging oligodendrocytes upregulate genes involved in ribosome biogenesis (*Rpl6, Rps29, and Rpl23a*) and immune-response (*C4b and Il33*) (Ximerakis et al., 2019; Rawji et al., 2023).

Adult rodent NG2 cells progressively loss their differentiation and proliferation potential which results in the slowing of remyelination capacity with aging (Neumann et al., 2019). They fail to be recruited into the lesion area and they are not able to differentiate into oligodendrocytes (Sim et al., 2002). This loss of function with aging is relevant for understanding that in chronically demyelinated MS lesions there is a decrease of the number of NG2 cells and their differentiation into oligodendrocytes (Neumann et al., 2019). Furthermore, NG2 cells microenvironment stiffens with age, and this stiffness appears to be sufficient to cause age-related loss of function of NG2 cells (Segel et al., 2019). Bulk RNA sequencing analysis revealed that the decline in functional capacity is associated with hallmarks of stem cell aging, such as mitochondrial dysfunction, decreased metabolic function, inflammasome signaling, increased DNA damage, dysregulated nutrient sensing, autophagy, and the unfolded protein response (Neumann et al., 2019; Rawji et al., 2023). Proteomic analysis reveals that the amount of myelin-associated proteins, and proteins associated with oxidative phosphorylation, inflammatory responses and actin cytoskeletal structure are upregulated by aging (de la Fuente et al., 2020). By contrast, enzymes related with transcription factors, cell cycle proteins, and biosynthesis of cholesterol, essential for the production of myelin, are downregulated (de la Fuente et al., 2020). Interestingly, regenerative capacity of aged NG2 cells can be enhanced with youthful systemic milieu containing monocytes, fasting, metformin treatment, or reduction of stiffness of the extracellular matrix. All these stimuli will improve remyelination in aged animals (Neumann et al., 2019; Segel et al., 2019).

#### 1.2.3. Astrocytes

Astrocytes, not only support neurons, but serve also a wide range of functions that involve ion buffering, water and ion homeostasis, neurotransmitter recycling, formation, maturation, maintenance, pruning and remodeling of synapses, blood–brain barrier formation, inflammation regulation, and interoception. They are located in close contact with all nervous system structures to accomplish metabolic and homeostatic functions indispensable for the proper functioning of neuronal circuits in the brain. They extend processes that occupy non-overlapping domains, into the vicinity of synaptic clefts. They enwrap presynaptic and postsynaptic neuronal regions, forming the so called “tripartite synapse.” In response to synaptic activity (Gomez-Gonzalo et al., 2017) or specific stimuli like hypercapnic acidosis in the brainstem (Beltran-Castillo et al., 2017), they show a calcium dependent release of gliotransmitters (Glu, Gaba, D-serine, ATP) which regulate the excitability and the efficacy of synapses (Araque et al., 2014). Since one astrocyte can contact thousands of synapses, they could recruit and enhance the activity of distant neurons and synapses in brain circuits (Halassa and Haydon, 2010). Together with microglia, astrocytes contribute also to inflammatory processes (Giovannoni and Quintana, 2020).

Astrocyte phenotype changes in response to brain injury, ischemia, infection, neuroinflammation or neurodegenerative diseases, which leads to “reactive astrocytosis.” Reactive astrocytosis involves morphological changes such as hypertrophy, increased astrocyte proliferation, up- and down-regulation of several genes, and particularly, after acute CNS trauma, are associated with glial scar formation (Liddelow et al., 2017; Lopez-Teros et al., 2022). Reactive astrocytosis englobes different astrocyte responses depending on the nature of insults (Zamanian et al., 2012). For example, neuroinflammation and stroke ischemia in mice result in upregulation of genes, whereas 50% are upregulated by both insults, the rest of genes are specific for each injury type. In brain inflammation, reactive astrocytes upregulate complement cascade which are deleterious for synapses, and compatible with a neurotoxic phenotype. By contrast, in ischemia, reactive astrocytes upregulate many neurotrophic factors suggesting a neuroprotective phenotype (Zamanian et al., 2012; Liddelow et al., 2017). As observed in cytotoxic microglia,. inflammatory astrocytes lose several astrocyte properties (Kigerl et al., 2009; Zamanian et al., 2012; Liddelow et al., 2017) such as promotion of neuronal survival, outgrowth, synaptic functions, and phagocytosis. They can induce death of neurons and oligodendrocytes *in vitro*, and of axotomized neurons *in vivo* (Liddelow et al., 2017). Increased numbers of activated astrocytes are also observed in human neurodegenerative diseases (Liddelow et al., 2017).

As part of astrocyte/microglia interaction, microglia modulate astrocytes (Liddelow et al., 2017). In wild-type (WT) mice, LPS injection activates astrocytes, whereas in *Csf1r*−/− mice, which lack microglia cells, LPS fails to the activation of astrocytes (Liddelow et al., 2017). Furthermore, activated microglia cells induce differentiation of astrocytes by secreting interleukin 1α (IL1α), tumor necrosis factor α (TNFα), and complement 1q (C1q), which together are necessary and sufficient to induce the activation (Liddelow et al., 2017). It is worth noting that C1q is part of the first component of the C1 complex, which is bound by antigen/antibody complexes, neuronal blebs, apoptotic cells, fibrillary β amyloid (Aβ) or phospho-tau, to activate the classical pathway of complement. C1q appears to have a role of tagging weak synapses to be engulfed by microglia (Gomez-Arboledas et al., 2021).

Reactive astrocytes can lead to beneficial or detrimental effects. For instance, they can produce a glial scar as a defensive barrier against inflammatory cells or pathogens, promote neuronal survival, detect signals of brain damage, or secrete cytokines and chemokines. However, they also can inhibit cell migration and axonal regeneration (Escartin et al., 2021; Lopez-Teros et al., 2022). The astrocyte’s responses to brain aging are heterogenous. Proliferation-competent glial cells can undergo senescence both *in vitro* and *in vivo*, and contribute to neuroinflammation in the aging brain. Astrocytes can become reactive or senescent with aging, depending on stressful stimuli, contributing to the loss of cognitive function through inflammatory mediators (Cohen and Torres, 2019; Escartin et al., 2021; Lopez-Teros et al., 2022; Gaspar-Silva et al., 2023). Astrocyte senescence implies permanent cell cycle arrest, increased cell size, and several characteristics that involve a secretory profile called senescent associated secretory phenotype (SAPS) (Rodier and Campisi, 2011), the presence of DNA damage or “scars,” changes in heterochromatin called Senescence-Associated Heterochromatin Foci (SAHF) (Narita et al., 2003), increment of b-galactosidase enzyme activity (Dimri et al., 1995), lipofuscin accumulation, and a decrease in lamin B1 (Shimi et al., 2011).

In humans, astrocyte- and oligodendrocyte-specific genes, but not neuron-specific genes, show significant age-dependent changes in their regional expression patterns, particularly in the hippocampus and substantia nigra. By contrast, microglia- and endothelial-specific genes increase in all brain regions (Soreq et al., 2017). A hallmark of brain physiological aging, the increase in astrocyte reactivity, assessed through the increase in glial fibrillary acidic protein (GFAP) labeling, is mainly observed in the hippocampus, frontal, temporal, and entorhinal cortex in humans (Nichols et al., 1993; Porchet et al., 2003). Similar findings have been observed in aged mice and rats (Nichols et al., 1993; Rodriguez et al., 2014; Boisvert et al., 2018). In mice, astrocytes undergo various age-dependent morphological and molecular changes in specific brain regions (Boisvert et al., 2018). Such changes involve astrocyte hypertrophy, rearrangement of cytoskeleton, GFAP upregulation, and inflammatory phenotype. Aged astrocytes from the hypothalamus and cerebellum, but not from the cortex, show significant increase in the expression of genes associated with inflammatory response, astrocyte reactivity (*GFAP* and *Serpin3n*), synapse elimination pathways (complement *C3 and C4b*), and downregulation of cholesterol synthesis enzymes (Boisvert et al., 2018).

RNA sequencing (RNAseq) analysis of the differentially expressed astrocytes genes along the mouse lifespan revealed that in aged astrocytes there is a significant increase in the proportion of reactive astrocytes exhibiting a neuroinflammatory phenotype (Clarke et al., 2018) in a brain region-dependent phenomenon. Hippocampal and striatal astrocytes upregulate a higher number of reactive astrocyte genes than that observed in cortical astrocytes. Furthermore, the LPS-induced transformation of astrocytes is increased by aging. Likely, the aging effect may be associated to the 300-fold increase in C1q observed in aging mouse and human brains (Stephan et al., 2013) and the upregulation of inflammatory genes in aging microglia, in special because the aging-dependent upregulation of reactive astrocyte genes was significantly reduced in mice lacking microglia-secreted cytokines (IL1α, TNF, and C1q) (Clarke et al., 2018).

These results show that microglia play a role in astrocyte activation. Aged astrocytes contribute to create an environment favoring synapse elimination and neuronal damage, likely promoting aging-associated cognitive decline (Boisvert et al., 2018). In fact, astrocytes undergo age-dependent changes in gene expression that make specific brain regions more vulnerable to age-associated synapse loss and neuroinflammation. If astrocyte heterogeneity defines the susceptibility of different brain regions to certain insults, and the way brain regions age are still open questions.

#### 1.2.4. Microglia

In contrast to neurons and astrocytes, microglia have mesodermal origin. Microglia are derived from myeloid precursor cells from the yolk sac, which differentiate in tissue-resident macrophage precursors that migrate into the CNS where they finally become resident microglia at an early embryonic state.

The microglia, as “resident macrophages” (Rivest, 2009), constitute the main defense system of the CNS (Tremblay et al., 2011). Microglia are the main contributors to synaptic pruning in the brain, although astrocytes can participate in this process, particularly, during development and early postnatal life. In adult animals, failure in microglia regulation of pruning can exacerbate synaptic loss leading to memory deficits observed in neurodegenerative diseases (Wu et al., 2015; Hong et al., 2016; Liddelow et al., 2017; Gomez-Arboledas et al., 2021).

Microglia surveil systematically the nervous tissue and detect a wide spectrum of signals associated with autoimmune damage, infections, ischemia, trauma, and toxins (Rivest, 2009). Depending on the nature of these signals, they can trigger an integrative microglial response to maintain brain homeostasis, modifying their morphology and functional properties, phagocytizing, and degrading potentially harmful endogenous and exogenous compounds. Thus, surveillance microglia can adopt an activated phenotype, which can be pro- or anti-inflammatory. Depending on their phenotype, microglia are able to release a broad spectrum of molecules including inflammatory cytokines, such as interleukin 1β (IL1β), interleukin 6 (IL6), tumor necrosis factor α (TNFα), and interferon γ (IFNγ), reactive oxygen species (ROS) (Qin et al., 2005; Block et al., 2007; Li et al., 2007; Kettenmann et al., 2011; Welser-Alves and Milner, 2013; Hickman et al., 2018), and nitric oxide (NO) (von Bernhardi and Eugenin, 2004; Li et al., 2007; Nakajima et al., 2007; Sierra et al., 2007; Tichauer and von Bernhardi, 2012; Welser-Alves and Milner, 2013; von Bernhardi et al., 2015). Interestingly, microglia can modulate regulatory cytokines such as interleukin 10 (IL10) and transforming growth factor β (TGFβ), among other trophic factors. In addition to be the main source of inflammatory molecules, they also release gliotransmitters (Glu, ATP and D-serine) in the CNS (Imura et al., 2013; Beltran-Castillo et al., 2018; Dos-Santos-Pereira et al., 2018).

Microglia undergo senescence, showing characteristic morphological and functional features (Paolicelli et al., 2022), that differ from those found in activated or quiescent microglia (Hart et al., 2012; Spittau, 2017; Wendimu and Hooks, 2022). A feature of abnormal aged dystrophic microglia is the reduction or absence of cytoplasmic processes leading, occasionally, to the formation of spheroids, a striking sign of dystrophia with partial fragmentation of their cytoplasm (Mecca et al., 2018; Edler et al., 2021; Javanmehr et al., 2022; Munoz-Castro et al., 2022; Paolicelli et al., 2022; St-Pierre et al., 2022). Double immunofluorescence, against ionized calcium binding adaptor molecule 1(Iba1)/cluster of differentiation 68 (CD68) and Iba-1/major histocompatibility complex (MHC) class II to identify microglia, together with electron microscopy, revealed that isolated Iba-1(+) fragments persisted connected to each other by CD68(+) or MHCII(+) segments of the microglial process. That is, apparent fragments by light microscopy, indeed still are connected to the microglial soma when observed by electron microscopy (Tischer et al., 2016).

Since microglia is the main generator of inflammatory cytokines and oxidative mediators in the CNS (Pawate et al., 2004; Qin et al., 2005; Hayashi et al., 2008), changes in the regulation of aged microglia may lead microglial activation from neuroprotective to deleterious (von Bernhardi et al., 2015). Hence, microglial dysregulation arises as a key element for inducing chronic neuroinflammation (Tremblay et al., 2011). On the other hand, neuroinflammation promotes neurotoxicity by inducing cytotoxic activation of microglia (Ramirez et al., 2008), inhibition of Aβ clearance (von Bernhardi, 2007), and synergistic deleterious effects promoting neuronal death (Nguyen et al., 2002; von Bernhardi and Eugenin, 2012). Thus, as a global effect of aging, microglia switch from a neuroprotective to a more cytotoxic phenotype (Banati et al., 1993; von Bernhardi et al., 2010, 2015, 2019; Streit et al., 2021). Accordingly, microglia from older individuals present morphological evidence of activation compared with young ones (Streit et al., 2004; von Bernhardi, 2007; von Bernhardi et al., 2010, 2011), elevated basal levels of inflammatory cytokines, such as IL6 and IL1β (Ye and Johnson, 1999; Sierra et al., 2007), decreased signaling by suppressor of mothers against decapentaplegic 3 (Smad3)-TGFβ in inflammation (Tichauer and von Bernhardi, 2012), decreased Aβ-induced phagocytosis (Floden and Combs, 2011), and increased ROS production (Tichauer and von Bernhardi, 2012) and oxidative stress (von Bernhardi and Eugenin, 2012; Tichauer et al., 2014). This microglia activation has been described in the aging CNS (von Bernhardi, 2007; von Bernhardi et al., 2010; Villeda et al., 2014), as well as, in various pathologies, including cerebrovascular disease and AD (Hickman et al., 2008; Murgas et al., 2012; von Bernhardi and Eugenin, 2012; Trougakos, 2019). Changes in the expression of receptors relevant for cellular communication and inflammatory activation (Gu et al., 2019) underly microglial dysfunction (Tarkowski et al., 2002; Tse and Herrup, 2017).

Microglial toxicity is modulated by astrocytes through a cross-regulation that includes TGFβ and IL1β (Tichauer et al., 2007; Orellana et al., 2013), the neuroprotective response being especially conspicuous in acidic microenvironments (Uribe-San Martin et al., 2009). Hypercapnic acidification has also effects on phagocytosis (Eugenin et al., 2016) and synaptic function, inducing the release of D-serine by astrocytes (Beltran-Castillo et al., 2017, 2018), which could also participate in aging. Multiple changes associated with glial dysregulation in aging (von Bernhardi, 2007; Hickman et al., 2018) result in the impairment of neuronal function, including the production of soluble mediators by activated cells in aging (Conboy et al., 2005, 2013; Villeda et al., 2014), which affect synaptic function (Johnson-Venkatesh and Umemori, 2010; Schafer et al., 2013; Cerpa et al., 2016), and the regulation of cellular activation by TGFβ and mTOR, the mammalian target of rapamycin (Flores and von Bernhardi, 2012; Herrera-Molina et al., 2012; Switon et al., 2017).

The development of methodology to cultivate microglia from aged mice (von Bernhardi et al., 2011), allowed us to determine that Aβ phagocytosis declines in microglia obtained from adult and aged mice (Alarcon et al., 2005; Cornejo and von Bernhardi, 2013). The reduced uptake is related to the decreased expression of Scavenger Receptor A (SRA), which is relevant for Aβ phagocytosis and appears to mediate the enhancement of Aβ cytotoxicity (Murgas et al., 2012), and for shaping glial inflammatory activation by regulating the secretion of cytokines, ROS and reactive nitrogen species, and the activation of signaling pathways associated with inflammation (Godoy et al., 2012; Murgas et al., 2014). SRA expression is regulated by TGFβ-Smad signaling (Cornejo et al., 2018), which is altered in aging and AD (Tichauer et al., 2014).

Recently, it has been proposed that a subpopulation of microglia can have a protective effect in neurodegenerative diseases, in particular AD and MS (Keren-Shaul et al., 2017; Krasemann et al., 2017). Using single cell transcriptomics, Keren-Shaul et al. (2017) recognized a microglia sub-population, which was denominated disease associated microglia (DAM), also known as activated response microglia (ARM) or microglial neurodegenerative phenotype (MGnD) (Keren-Shaul et al., 2017; Sobue et al., 2023). DAM microglia were found near Aβ plaques in human and mice brains showing intracellular phagocytosed Aβ, suggesting their capacity to restrict Aβ plaque formation by degrading Aβ (Keren-Shaul et al., 2017). Physiologically, DAM activation is achieved via a 2-step process. The first step is a triggering receptor expressed on myeloid cells 2 (TREM2)- independent process, in which microglia transits toward to a stage 1 DAM, characterized by a reduced expression of homeostatic microglia checkpoint genes such as *Cx3cr1* and *P2ry12/P2ry12*, and upregulation of *B2m*, and AD-associated genes such as *Tyrobp* and *Apoe* (Xu et al., 2022). The second step is a TREM2-dependent process, in which stage 1 DAM cells are transformed into stage 2 DAM cells, characterized by the upregulation of *Cst7*, *Lpl*, and *Trem2* genes (Xu et al., 2022). It is thought that the inhibition of some of these microglia specific inhibitory checkpoints (such as *Cx3cr1*), which disinhibits DAM activation, could be an important therapeutic target (Keren-Shaul et al., 2017). Another study identified a similar subpopulation of microglia, named neurodegeneration-associated microglia (MGnD) controlled by TREM2-APOE pathway and post-transcriptionally regulated by microRNA(miR)-155 in the surrounding of plaques (Krasemann et al., 2017). TREM2 activates APOE pathway, which transforms a homeostatic into a neurodegenerative microglia phenotype after phagocytosis of apoptotic neurons. Interestingly, targeting the TREM2-APOE pathway can restore the homeostatic phenotype of microglia in amyotrophic lateral sclerosis (ALS) and AD mice models, and prevented neuronal loss in an acute neurodegeneration model (Krasemann et al., 2017).

### 1.3. The CX_3_CL1/CX_3_CR1 axis

**Figure 1:**
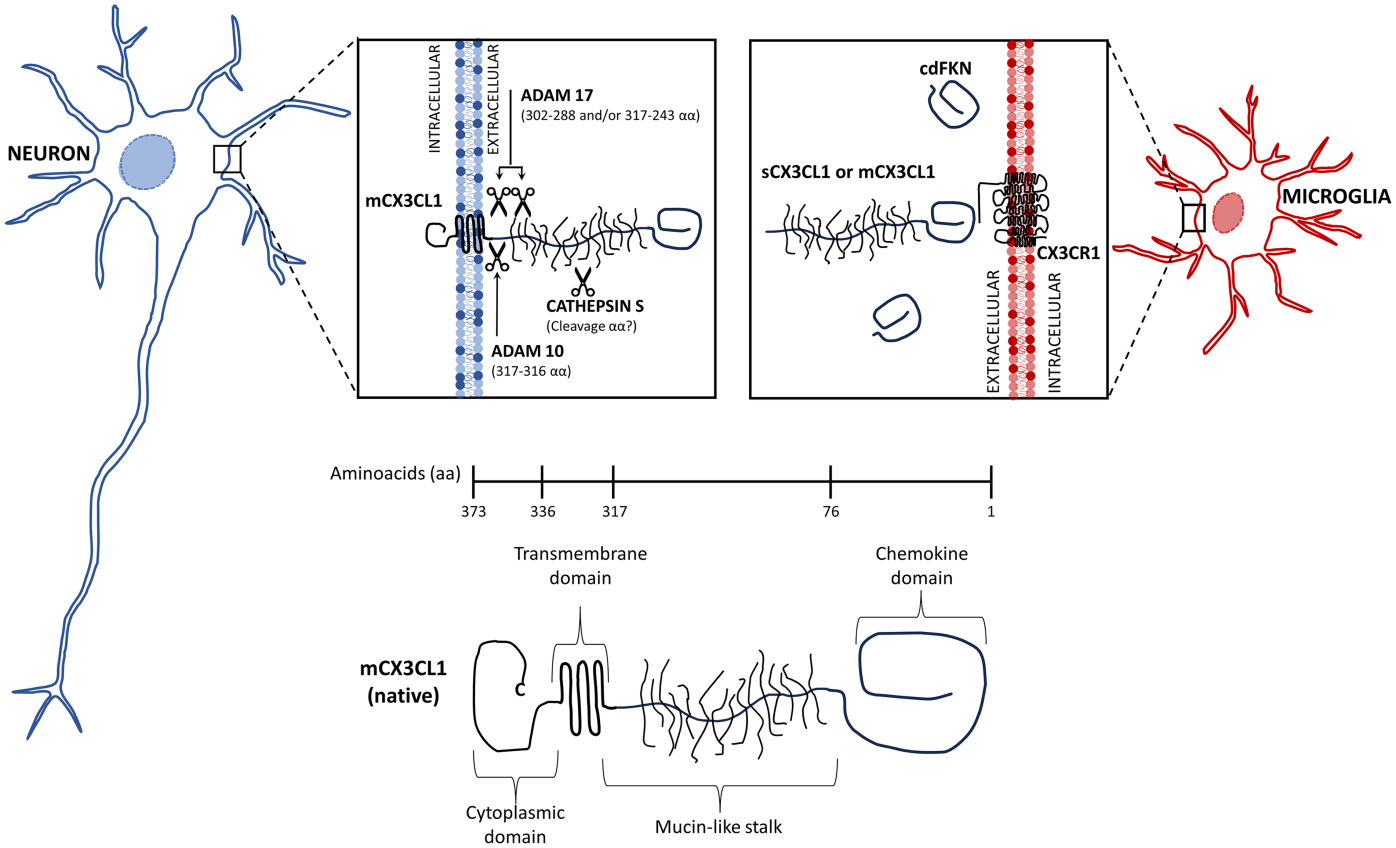
Membrane-bound and soluble fractalkine. Fractalkine (CX_3_CL1) is synthesized in neurons as a precursor that is rapidly transported to the cell surface, where it is incorporated as a transmembrane protein (mCX_3_CL1, native). We illustrate the mCX_3_CL1 molecule indicating its chemokine, mucine-like stalk, transmembrane, and cytoplasmic domains. At the cellular surface, mCX_3_CL1 is targeted for metalloproteinase-dependent cleavage that releases soluble fractalkine (sCX_3_CL1) containing most of the mucine-like stalk and the chemokine domain. Key proteases contributing to CX_3_CL1 cleavage include TNFα converting enzyme (TACE; ADAM17), ADAM10, and cathepsin S. Physiological CX_3_CL1 cleavage occurs at different sites depending on the protease. sCX_3_CL1 forms do not differ much in their functional effects. Both CX_3_CL1 types, mCX_3_C1L and sCX_3_CL1, bind the CX_3_CR1 located in microglia. A CX_3_CL1 form restricted to the chemokine domain (cdCX_3_CL1) has been artificially generated.

The chemokine fractalkine is found as an anchored transmembrane protein, with its chemokine domain bound to a mucin stalk, which endows it with a cell adhesion function (Bazan et al., 1997; Figure 1). CX_3_CL1 is composed of 373 amino acids (aa), that conform four domains: chemokine domain (CKD, 76 aa), mucin stalk domain (MS, 241 aa), containing 17 mucin repeats with glycosylation-dependent stiffness to present the CKD away from the membrane and reduce the CX_3_CL1 diffusion within the membrane, transmembrane domain (TM, 19 aa), involved in the aggregation of several CX_3_CL1 molecules to strengthen adhesion, and cytosolic domain (CD, 37 aa) that provides anchoring to cytoskeletal proteins increasing fractalkine adhesion with its receptor (Ostuni et al., 2014, 2020; Rivas-Fuentes et al., 2021; Figure 1).

In the CNS, CX_3_CL1 is abundant and constitutively expressed by neurons, whereas its expression by astrocytes is induced by TNFα and IFNγ (Yoshida et al., 2001). The CX_3_CL1 anchored to the neuron membrane is known as the “membrane bound CX_3_CL1” (mCX_3_CL1). However, in an inflammatory environment, it undergoes cleavage by metalloproteases (ADAM 10 and ADAM 17) and other proteases, releasing soluble forms (sCX_3_CL1) (Jones et al., 2010). All forms of fractalkine bind to the sole receptor CX_3_CR1, a 7-transmembrane receptor coupled to heterotrimeric G protein (GPCRs) found constitutively in microglia (Harrison et al., 1998; Williams et al., 2014; Szepesi et al., 2018), whereas in astrocytes it is expressed only under inflammatory conditions (Rivas-Fuentes et al., 2021).

The binding of CX_3_CL1 with CX_3_CR1 dissociates the α subunit from the βγ complex of the associated-G protein, activating several signaling pathways and intracellular Ca^2+^ mobilization (Harrison et al., 1998; Boddeke et al., 1999), phosphoinositide 3-kinase (PI3K) and mitogen activated protein kinases (MAPK), such as c-Jun N-terminal kinase (JNK), extracellular-signed regulated kinase (ERK) 1/2, p38-mitogen activated protein kinase (p38 MAPK), protein kinase B (PKB, also called AKT Ser-473 Thr-308), proto-oncogene tyrosine-protein kinase Src (c-Src), and endothelial nitric oxide synthetase (eNOS) (Meucci et al., 2000; Cambien et al., 2001; Kansra et al., 2001; Deiva et al., 2004; Lee et al., 2006; Volin et al., 2007; Yang et al., 2007), which contribute to cellular responses such as migration, survival and apoptosis resistance.

The CX_3_CL1/CX_3_CR1 axis contributes to brain functions along the whole lifespan. During development, CX_3_CL1/CX_3_CR1 affects survival, the maturation of neuronal networks, microglial recruitment and pruning (Paolicelli et al., 2011; Ueno et al., 2013), and functional maturation of synapses (Hoshiko et al., 2012). In the adult brain, CX_3_CL1/CX_3_CR1 signaling regulates glutamatergic synaptic transmission and plasticity (Bertollini et al., 2006; Ragozzino et al., 2006; Maggi et al., 2009), as well as cognitive functions (Maggi et al., 2011; Rogers et al., 2011; Sheridan et al., 2014).

Several experiments have been performed in mouse models that control the expression of fractalkine and its receptor: *Cx3cl1-mCherry*, in which exon 1 of *Cx3cl1* is replaced with an *mCherry* fluorescent protein. *mCherry* fluorescence is observed in mature neurons in the hippocampus, striatum, and cortical layer II and in epithelial cell layers. *Cx3cr1^+/GFP^* knock-in (Kin) mice, in which the *Cx3cr1* gene was replaced by a green fluorescent protein (GFP) reporter gene, that is, the heterozygote *Cx3cr1 ^+ /GFP^* mice express GFP in cells that retain receptor function; this Kin model allows to generate a *Cx3cr1*−/− mouse through a *Cx3cr1^GFP/GFP^* double Kin mouse model. *Cx3cr1-Cre* mice express Cre recombinase under the direction of the *Cx3cr1* promoter in the mononuclear phagocyte system. These mice do not express endogenous *Cx3cr1*. *Cx3cr1^CreER^* Kin/knock-out (KO) mice express a tamoxifen-inducible Cre recombinase under the direction of the *Cx3cr1* promoter in the mononuclear phagocyte system. Insertion of the Cre-ER fusion protein KO endogenous CX_3_CR1 expression.

### 1.4. CX_3_CL1/CX_3_CR1 axis and inflammation

*In vitro* and *in vivo* experimental results suggest that activation of the CX_3_CL1/CX_3_CR1 axis has anti-inflammatory effects. CX_3_CL1 inhibits the bacterial endotoxin lipopolysaccharide (LPS)-induced release of TNFα, IL6, and IL1β by microglia in culture (Zujovic et al., 2000; Mizuno et al., 2003). Furthermore, LPS-induced TNFα secretion was enhanced by CX_3_CL1 immune neutralization (Zujovic et al., 2000). Accordingly, neutralizing anti-CX_3_CL1 antibodies enhance acute brain inflammation induced by the intracerebroventricular (ICV) injection of LPS (Zujovic et al., 2001). In fact, *Cx3cr1−/−* mice show an increased microglial IL1β expression, neurotoxicity, and higher mortality induced by repeated intraperitoneal (IP) injections of LPS than those observed in *Cx3cr1+/−* mice (Cardona et al., 2006).

LPS treatment downregulates, in turn, the expression of CX_3_CR1 in rat and mouse microglia cell cultures (Zujovic et al., 2000; Inoue et al., 2021). Fractalkine applied before LPS, inhibits the increase of LPS-induced NO. The overexpression of CX_3_CR1 decreased the LPS-induced NO production even without the application of exogenous CX_3_CL1 (Inoue et al., 2021).

Similarly, in *in vivo* experiments, a reduction of CX_3_CR1 mRNA is observed 4 h after the IP injection of LPS in adult and aged mice microglia. The LPS-induced reduction of CX_3_CR1 mRNA is observed for up to 24 h on aged mice microglia. Interestingly, CX_3_CR1 downregulation was associated with a prolonged microglial activation, reduction of TGFβ, and persistent signs of sickness behavior (Wynne et al., 2010). Given that 4 h of TGFβ treatment of BV2 microglia increases CX_3_CR1 mRNA and decreases IL1β mRNA (Wynne et al., 2010), it appears that the CX_3_CL1/CX_3_CR1 axis and TGFβ pathway are under reciprocal regulation to generate a more intense anti-inflammatory response.

CX_3_CL1 treatment is also effective for attenuating inflammatory activation. Its protective effects have been reported on microglia activation and behavioral performance after radiation-induced brain injury (RIBI) (Wang et al., 2021). Irradiation of BV2 microglia in culture induces inflammatory activation and increase of pro-inflammatory cytokines mediated by activation of the microglial nuclear factor κB (NFκB) pathway (Xue et al., 2014; Dong et al., 2015). RIBI activates also microglia NFκB in mice, increasing mRNA and protein expression of TNFα and IL1β, associated with hippocampal neurogenesis impairment and memory deficit evaluated with Morris water maze, in which the mouse must localize a submerged platform guided by visual and spatial cues allowing to test memory, learning, and spatial working (Dong et al., 2015). Exogenous CX_3_CL1 reduced the RIBI-induced increase in IL1β and TNFα, facilitates inflammatory microglial to change into an anti-inflammatory phenotype, and improves the performance of irradiated mice in the Morris water maze (Wang et al., 2021).

**Figure 2:**
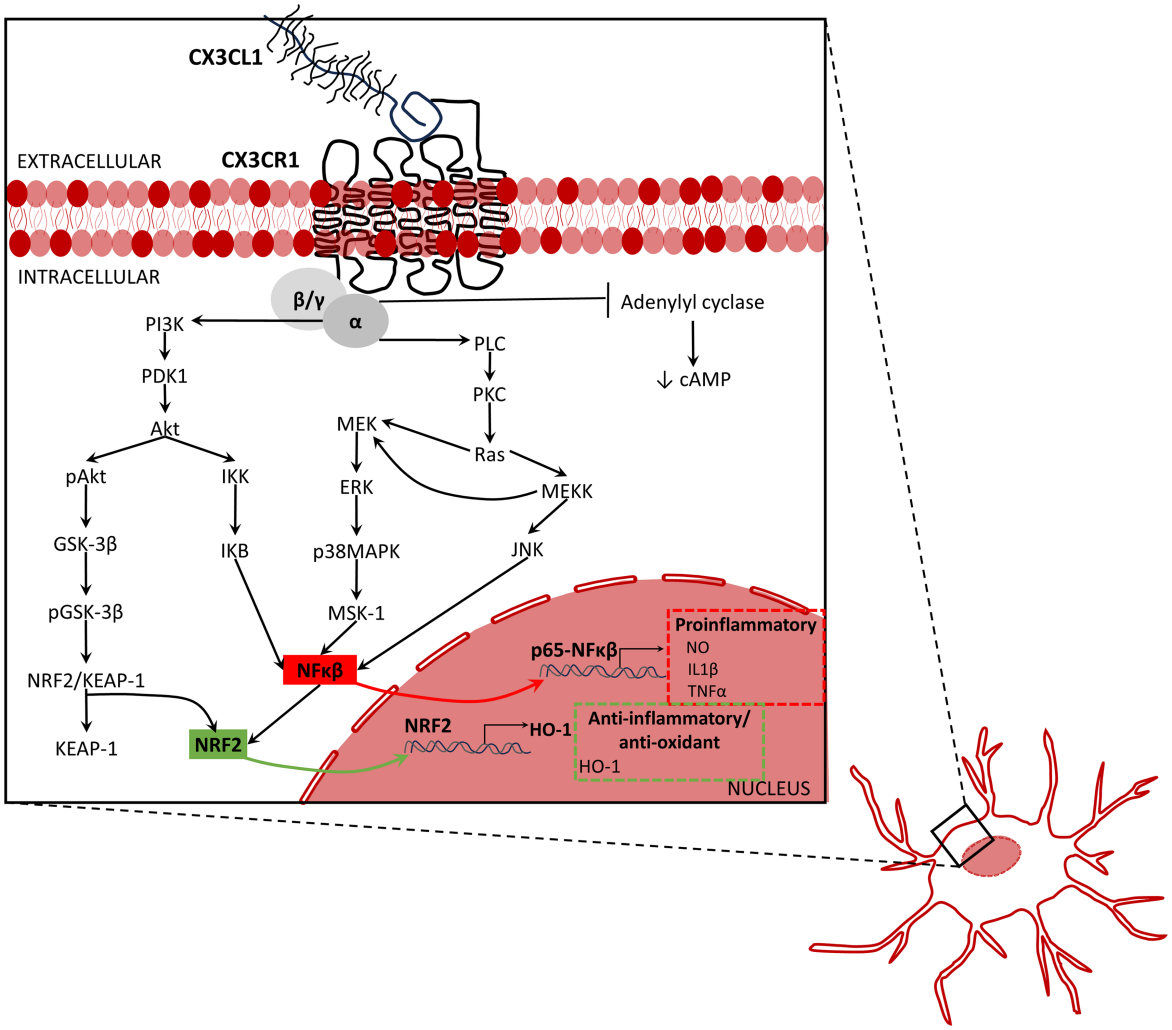
Inflammation associated signaling activated by fractalkine. The activation of the CX_3_CL1/CX_3_CR1 axis activates the mitogen-activated protein kinases (MAPKs) including the extracellular signal-regulated kinases (ERK), p38-MAPK, and c-Jun NH(2)-terminal kinase (JNK). MAPKS activate the stress-activated protein kinase-1 (MSK1), and consequently, activate NFκB pathway, and therefore, the production and release of inflammatory mediators. By contrast, the activation of the AKT/ERK signaling pathway induces factor 2 related to nuclear factor E2 (Nrf2) to be translocated into the nucleus leading to increased transcription of various antioxidant and cytoprotective genes such as antioxidant response element (ARE) and heme oxygenase-1 (HO-1), which increase the phagocytic and anti-inflammatory capacity of microglia. To summarize, activation of CX_3_CL1/CX_3_CR1 axis increases NFκB (Galan-Ganga et al., 2019; Liu et al., 2019), promotes release of pro-inflammatory cytokines by microglia, and enhances the Nrf2 activation that increases anti-oxidant and anti-inflammatory response (Lastres-Becker et al., 2014; Castro-Sanchez et al., 2019; Li et al., 2019; Trougakos, 2019). Akt, protein kinase B; ERK, extracellular signal-regulated kinases; GSK-3β, glycogen synthase kinase-3β; HO-1, heme oxygenase 1; IKB, Ikappa B protein; IKK, IkappaB kinase; IL1β, interleukin-1β; JNK, c-Jun N-terminal kinase; KEAP-1, Kelch-like ECH associating protein-1; MEK, mitogen-activated protein kinase kinase; MEKK, MEK kinase or mitogen activated protein (MAP) kinase kinase kinase; MSK-1, mitogen- and stress-activated kinase 1; NFκB, nuclear factor kappa-light-chain-enhancer of activated B cells; NRF2, nuclear factor erythroid 2-related factor 2; NRF2/KEAP-1, nuclear factor erythroid 2-related factor 2/Kelch-like ECH associating protein-1;NO, nitric oxide; pAkt, phosphorylated AKT; pGSK-3β, phosphorylated glycogen synthase kinase-3β; PDK1, protein 3-phosphoinositide-dependent protein kinase-1; PI3K, phosphoinositide 3-kinase, also called phosphatidylinositol 3-kinase; PKC, protein kinase-C; PLC, phospholipase C p38MAPK, p38 mitogen-activated protein kinase; Ras, rat sarcoma virus small-GTPase; TNFα, tumor necrosis factor-α.

The intracellular signaling pathways activated by CX_3_CL1/CX_3_CR1 axis affect neuroprotection. Activation of CX_3_CR1 activates MAPKs. Activation of ERK, p38-MAPK, and JNK, through the activation of the stress-activated protein kinase-1 (MSK1) (Bachstetter et al., 2011; Galan-Ganga et al., 2019), activate NFκB, which increases the expression of inflammatory mediators (Zujovic et al., 2000; Galan-Ganga et al., 2019; Figure 2). By contrast, the activation of the AKT/ERK signaling pathway induces factor 2 related to nuclear factor E2 (Nrf2) that translocate to the nucleus and increases the transcription of various antioxidant and cytoprotective genes such as antioxidant response element (ARE) and heme oxygenase-1 (HO-1), which increase the phagocytic and anti-inflammatory capacity of microglia (Lastres-Becker et al., 2014; Castro-Sanchez et al., 2019; Li et al., 2019; Trougakos, 2019; Figure 2). Hence, as a summary, activation of CX_3_CL1/CX_3_CR1 axis can activate NFκB (Galan-Ganga et al., 2019; Liu et al., 2019), increasing production of inflammatory cytokines by microglia, and enhance the Nrf2 activation, increasing antioxidant and anti-inflammatory effects (Lastres-Becker et al., 2014; Castro-Sanchez et al., 2019; Li et al., 2019; Trougakos, 2019).

Activation of CX_3_CL1/CX_3_CR1 does not lead always to neuroprotection, depending on the triggering inflammatory-like processes. After transient occlusion of the middle cerebral artery (MCAO), *Cx3cr1^GFP/GFP^* mice exhibited reduced areas of brain infarcts, reduced number of apoptotic cells and infiltrating leucocytes, and reduced blood–brain barrier (BBB) damage compared with *Cx3cr1^+/GFP^* and *Cx3cr1^+/+^* mice. Moreover, *Cx3cr1^GFP/GFP^* mice expressed less IL1β, IL1Ra, and TNFα mRNAs and showed better functional outcomes than the other groups (Denes et al., 2008). The disruption of the CX_3_CL1/CX_3_CR1 axis in permanent MCAO upregulate CX_3_CR1 in neurons in the striatum and the hippocampus, which was associated with an increased apoptotic-like neuronal morphology and increased number of caspase 3(+) neurons (Wang et al., 2018). By contrast, ischemia-induced apoptotic neuronal cell death was decreased in CX_3_CR1 KO mice (Wang et al., 2018). That is, CX_3_CR1 deletion can endow neuroprotection in mouse models of brain ischemia (Denes et al., 2008; Wang et al., 2018).

The CX_3_CL1/CX_3_R1 axis is regulated by inflammatory cytokines such as TGFβ, IFNγ, TNFα, or IL1β (Rivas-Fuentes et al., 2021). In newborn rat microglia cultures, 1–10 ng/mL TGFβ1 for 16 h increased CX_3_CR1 mRNA and protein level (Chen et al., 2002), and reduce the activation of microglial ERK1/2 and p38 MAPK induced by a 10 min CX_3_CL1 stimulation (Chen et al., 2002).

### 1.5. The impact of CX_3_CL1/CX_3_CR1 axis on neurodegenerative diseases

Although, the absence of CX_3_CR1, *per se*, does not result in microglia activation or neurodegeneration, the impairment of the CX_3_CL1/CX_3_CR1 signaling by deletion of the *Cx3cr1* gene results in increased neurotoxicity in mouse models of LPS-induced systemic inflammation, Parkinson’s disease (PD), AD, and ALS (Cardona et al., 2006; Lee et al., 2010; Cho et al., 2011). Conversely, activation of CX_3_CL1/CX_3_CR1 axis can promote neural protection not only in different inflammatory conditions but also in pathological processes leading to neurodegenerative diseases and their associated inflammation.

#### 1.5.1. Amyotrophic lateral sclerosis

In murine model of ALS, in which human superoxide dismutase 1 (SOD1) with the G93A mutation is expressed under control of the cistronic human SOD1 promotor, the lack of expression of CX_3_CR1 accelerates ALS-like disease progression leading to a rapid and increased neuronal cell death (Boillee et al., 2006; Cardona et al., 2006; Liu et al., 2019). In this murine model of ALS, the absence of CX_3_CR1, increases the activation of NFκB, and impairs the autophagy-lysosome degradation pathway and the autophagosome maturation (Liu et al., 2019) resulting in an intense damage of motoneurons. Thus, the CX_3_CL1/CX_3_CR1 pathway has anti-inflammatory and neuroprotective effects and appears to play an important role in maintaining autophagy activity.

The *Cx3cr1* human polymorphism I249/M280 is present in about 20% of the population. The protein product of this polymorphism exhibits reduced affinity for CX_3_CL1 resulting in a reduced regulatory effect on microglia. Accordingly, the hypofunctional variant of CX_3_CR1 has been associated with a reduction of the life span of ALS patients (Lopez-Lopez et al., 2014). Thus, ALS patients, who carry one or two copies of the *CX3CR1-Val249Ile* allele, have a rapid course of the disease and a shorter survival than patients who are carriers of the WT *Cx3cr1* (Lopez-Lopez et al., 2014).

#### 1.5.2. Multiple sclerosis

MS is a chronic, inflammatory, predominantly immune-mediated disorder of the CNS showing focal lesions in white matter of the brain and spinal cord, characterized by prominent demyelination ensuing axonal damage and glial scar formation, leading to loss of motor and sensory function (Karussis, 2014; Oh et al., 2018; Dobson and Giovannoni, 2019; Absinta et al., 2020). In MS patients, *Cx3cr1-Val249Ile* polymorphisms revealed that *Cx3cr1 Ile249 Thr280* haplotype could endow a protective effect by impairing the switch of MS from the relapsing–remitting type (RRMS) into the secondary progressive type (SPMS) (Stojkovic et al., 2012; Arli et al., 2013). Patients having the variant in both alleles (homozygosity) have a higher risk for disability (Arli et al., 2013).

Current immune therapy is useful for ameliorating RRMS, but not for preventing its progressive forms (Oh et al., 2018; Dobson and Giovannoni, 2019; Absinta et al., 2020). Among several processes, remyelination is required for normalization of neural functions (Kotter et al., 2011; Lampron et al., 2015; Rivest, 2015; Mendiola et al., 2022). Unfortunately, remyelination in MS fails or is incomplete (Oh et al., 2018; Dobson and Giovannoni, 2019).

Chronic microglia activation is related with MS disease progression (Absinta et al., 2020). Besides, a switch in microglia from a pro-inflammatory to a regulatory phenotype is associated with the beginning of remyelination (Miron et al., 2013). The regulatory phenotype favors oligodendrocyte differentiation and is observed in injured CNS regions of aged mice in which remyelination is enhanced, like in MS lesions (Miron et al., 2013).

It has been suggested that CX_3_CL1/CX_3_CR1 axis is a relevant regulator of the clearance of myelin debris by microglia (Lampron et al., 2015; Rivest, 2015; Mendiola et al., 2022). Experimental autoimmune-independent demyelination without BBB disruption followed by complete remyelination can be attained with cuprizone, a copper chelating toxin that induces apoptosis of oligodendrocytes and can be administered through the diet (Lampron et al., 2015; Rivest, 2015; Mendiola et al., 2022). In WT mice, cuprizone induces massive demyelination as consequence of oligodendrocytes apoptosis during the first 3 weeks (wk.) accompanied by recruitment of NG2 cells, astrogliosis, and microgliosis (Hiremath et al., 1998; Gudi et al., 2014). Cuprizone removal from diet is followed by complete remyelination within 1–3 wk. In CX_3_CR1^−/−^ mice, inflammatory response was like that observed in cuprizone-challenged WT mice. However, cuprizone-induced demyelination was followed by uncomplete remyelination. Microglia in CX_3_CR1^−/−^ mice exhibited an impaired migration into the *corpus callosum* and phagocytosis, and consequently, persistent myelin debris and defective axonal remyelination characterized by aberrant myelin patterns. Unlike WT microglia that showed abundant phagocytic inclusions, CX_3_CR1^−/−^ microglia lack them (Lampron et al., 2015). Induction of experimental autoimmune encephalomyelitis (EAE) on a mouse model of CX_3_CR1 hypofunction by replacement of the normal mo *Cx3cr1* locus for the hu *Cx3cr1-I249/M280* variant, revealed exacerbated functional signs of EAE, with more severe inflammation and neuronal loss (Cardona et al., 2018).

Transgenic mice expressing the polymorphic hu *Cx3cr1-I249/M280* variant, and fractalkine-deficient (*Cx3cl1^−/−^*) mice showed exacerbated cuprizone-induced demyelination in the anterior corpus callosum 4 w, compared with that observed in CX_3_CR1^−/−^ deficient (*Cx3cr1^GFP/GFP^*) and WT mice (Mendiola et al., 2022). Microgliosis and CD68 (phagocytic microglia marker) were similar in hu *Cx3cr1-I249/M280, Cx3cl1^−/−^*, and *Cx3cr1^GFP/GFP^* mice, but higher than those in WT mice after cuprizone treatment (Mendiola et al., 2022). Notoriously, after 1 wk. removal of cuprizone, only *Cx3cr1^GFP/GFP^* and WT mice showed significant remyelination (Mendiola et al., 2022). WT mice showed significant remyelination at the anterior and posterior corpus callosum. In hu *Cx3cr1-I249/M280* mice, significant remyelination was only observed in the anterior corpus callosum, whereas in *Cx3cr1^GFP/GFP^* mice, only in the posterior corpus callosum. By contrast, *Cx3cl1^−/−^*mice did not show significant early remyelination (Mendiola et al., 2022).

Using EAE as a rat model of MS, it was shown that 12 days after the inoculation of myelin basic protein (MBP), CX_3_CL1 and CX_3_CR1 mRNA and protein levels were increased in dorsal root ganglia (DRG) and spinal cord (Zhu et al., 2013). The increase was associated with thermal sensory abnormalities, suggestive of neuropathic pain, and correlated with neurological impairment (Zhu et al., 2013). These results are indicative of the role of CX_3_CL1/CX_3_CR1 axis as a critical pathway involved in the MS-induced neuropathy.

#### 1.5.3. Retinal neurodegeneration

Studies in humans of the genotype distribution of CX_3_CL1 variants and their association with disease, also show a potential link between CX_3_CR1-Thr280Met, a variant associated with cell migration deficit, and a higher risk of human age-dependent macular degeneration (AMD) (Tuo et al., 2004; Chan et al., 2005; Combadiere et al., 2007). Evidence supporting a role for CX_3_CR1-Val249Ile variant in AMD is controversial (Combadiere et al., 2007). Homozygosity for the Thr280Met allele is a more consistent finding in AMD than of other diseases (Combadiere et al., 2007). In the macula of AMD, photoreceptors show signs of degeneration, retinal pigment epithelium is disrupted and cells expressing CX3CR1 are found in the outer retina, in the subretinal space, in the perivascular vicinity, and in choroidal neovascularization. In addition, deposits of CX_3_CR1 were found in drusen spots (Combadiere et al., 2007). Because they show a reduced affinity for CX_3_CL1, deletion of CX_3_CR1 has been experimentally used to simulate the main effect of these variants. Interestingly, aged albino mice with *Cx3cr1* deletion exhibit, in regions of retinal degeneration, accumulation of microglia in the subretinal space associated with drusen-like yellowish-white dots, and increased choroidal neovascularization, histological findings like those found in patients with AMD (Combadiere et al., 2007).

Retinitis pigmentosa is the more common non-syndromic inherited retinal dystrophy that include several heterogeneous retinal neurodegenerative conditions in which mutations in photoreceptor or retinal pigment epithelium genes result in progressive degeneration of photoreceptors (Hartong et al., 2006; Murro et al., 2023). Retinitis pigmentosa is characterized by a primary degeneration of rod photoreceptors that progresses to the loss of cone photoreceptors, followed by an aberrant remodeling of retina resulting in disconnection of neural retina from photoreceptors, associated with changes from the molecular to tissue levels (Hartong et al., 2006; Zieger et al., 2014; Murro et al., 2023).

Retinal degeneration 10 (rd10) is a mouse model of autosomal recessive retinitis pigmentosa, consisting in the spontaneous mutation of the rod-phosphodiesterase-6b (*Pde6b*) gene, that leads to rod degeneration and later to cone degeneration (Chang et al., 2002). Analysis of mRNA and protein levels of CX_3_CL1 revealed that in both WT and rd10 mice, a 100 kDa membrane bound CX_3_CL1 form and a cleaved soluble 85 kDa CX_3_CL1 form were present at the postnatal day 5 (P5). At P10, a 95 kDa form is accumulated, whereas the 85-kDa form is decreased. In older animals, the 95 kDa form became principal in wt retina, whereas in rd10 retinas, there is a significant increase of soluble 85 kDa form. Retinas of rd10 mice had significantly lower levels of total CX_3_CL1 protein (from P10 onwards) and lower CX_3_CL1 mRNA levels (from P14) than those observed in WT animals. *In situ* hybridization histochemistry and immunofluorescence using transgenic *Cx3cl1cherry* mice showed that neurons of the inner retina layers were the main sites of fractalkine synthesis in WT and rd10 mice (Zieger et al., 2014). In the rd10 mouse model, CX_3_CL1 was detected in apoptotic photoreceptors, suggesting a potential role of this chemokine on the recruitment of microglia into the outer nuclear layer of the retina (Makabe et al., 2020).

Microglia have been implicated in many degenerative eye disorders, including retinitis pigmentosa (Wang and Cepko, 2022), in which activated microglia phagocytose death photoreceptors and their debris and release pro-inflammatory factors that contribute to retinal degeneration (Zabel et al., 2016). In fact, in CX_3_CR1 deficient (*Cx3cr1^GFP/GFP^*) rd10 mice, microglial infiltration into the photoreceptor layer was greater, and the photoreceptor atrophy and apoptosis were higher than those observed in heterozygous *Cx3cr1^GFP/+^* rd10 littermates. Furthermore, CX_3_CR1 deficient microglia showed increased phagocytosis and increased expression of inflammatory cytokines associated with the increase of activation markers. Activation of CX_3_CL1/CX_3_CR1 axis in the rd10 retina via exogenous intravitreal delivery of recombinant CX_3_CL1 decreased microglial infiltration, phagocytosis, and activation together with reduction of morphological damage and improvement of photoreceptors functionality (Zabel et al., 2016).

Furthermore, synthetic progestin ‘norgestrel’ has been proven as a neuroprotective agent in the retinitis pigmentosa, likely throughout the increase of growth factors, such as basic fibroblast growth factor (bFGF) and leukemia inhibitory factor (LIF) in the retina, favoring the upregulation of pro-survival and downregulation of apoptotic pathways and reduction of microglial pro-inflammatory activity (Doonan et al., 2011; Roche et al., 2016). In primary cultures, rd10 microglia promote neuronal cell death. Norgestrel, in contrast, reduce pro-inflammatory activation and prevent neuronal cell death, reducing the expression of cytokine, chemokine, and danger-associated molecular pattern molecule (DAMP) in the rd10 retina. Furthermore, norgestrel upregulates CX_3_CL1/CX_3_CR1 signaling in the rd10 mouse, 1,000-fold at the RNA level (Roche et al., 2016). Norgestrel’s neuroprotection would be mediated by its actions on photoreceptors, which are induced to release CX_3_CL1, and this chemokine in turn, would refrain harmful microglia activity (Roche et al., 2017).

#### 1.5.4. Alzheimer’s disease

Alzheimer’s disease (AD) is a progressive neurodegenerative disorder and the main cause of dementia. Clinical symptoms include memory loss, learning difficulties, and problem-solving impairment. The main risk factor is aging; approximately 5–8% individuals older than 65 are affected, increasing to 35–50% in adults older than 85. The prevalence of AD for women is higher than in men by 19%. Risk factors associated with development of AD involve genetic predisposition (familial early-onset forms), allele ApoE-4 for apolipoprotein E, age, sedentarism, hypertension, diabetes, and metabolic syndrome, among others.

AD is characterized by the extracellular accumulation of Aβ_1-42_ (senile plaques) and intraneuronal aggregates as neurofibrillary tangles (NFTs) of hyperphosphorylated microtubule associated protein tau (MAPT) (Jellinger, 2020). The time course of AD progression indicates that Aβ deposition begins around 25 years before the onset of clinical diagnoses of AD, and is followed by neurofibrillary tangles (NFT) formation (Saito and Saido, 2018; Jellinger, 2020; Yin et al., 2021). Aβ is generated from the amyloid precursor protein (APP) cleavage by a two-step process: the β-site APP cleaving enzyme 1 (BACE1, or β-secretase), cleaves APP releasing a globular extracellular soluble protein and the membrane-anchored C-terminal fragment; later, γ-secretase (Presenilin) subsequently cleaves this fragment to excise Aβ (a peptide mostly of 39–43 amino acids). In addition to the protein aggregates, AD is characterized by the activation of microglia and astrocytes, neuroinflammation, and the progressive degeneration of neurons, with a predominant early damage of the entorhinal cortex and the hippocampus.

The participation of the CX_3_CL1/CX_3_CR1 axis on the course of AD differ in different AD mouse models. In APP/PS1 mice, a well-known mouse model for AD, the level of CX_3_CR1 does not differ, but the level of CX_3_CL1 is slightly reduced compared with WT littermates. In APP/PS1 mice, *Cx3cr1* deletion reduces plaques, either because an increased clearance or a reduced deposition of Aβ plaques. However, rTg4510 mice, an AD model with inducible overexpression of human mutant tau (P301L), show a 5-fold increase in CX_3_CR1 and a slight increase in CX_3_CL1 levels compared with the WT littermates (Nash et al., 2013). In the hu Tau model for AD, *Cx3cr1^−/−^* accelerates the onset of tauopathy and behavioral deficits (Bhaskar et al., 2010; Lee et al., 2010; Cho et al., 2011). Furthermore, the increased presence of sCX_3_CL1 ameliorates the severity of tauopathy in rTg4510 mice. Three months before rTg4510 mice show significant neuron loss, mice were injected with recombinant adeno associated virus (rAAV), expressing sCX3CL1, into both hippocampi. Three months after viral injection, 6 months old mice expressing sCX_3_CL1 show a significant decrease in tau pathology, an increased density of NeuN(+) cells, reduced hippocampus volume loss, and reduced microglia compared with the control condition (Nash et al., 2013). Memory and learning performance of WT and rTg4510 mice were assessed in the radial arm water maze. The injection of rAAV coding for sCX_3_CL1 did not significantly improve the behavioral deficit observed in the rTg4510 mice compared with non-transgenic mice (Nash et al., 2013).

To reveal the effect of CX_3_CL1/CX_3_CR1 axis on Aβ-induced toxicity, neuron–microglia primary cultures were exposed to hu Aβ_1-42_. WT neurons released CX_3_CL1 in response to hu Aβ_1-42_. Administration of 2 μM hu Aβ_1-42_ in microglia-depleted *Cx3cr1^−/−^* mixed cortical and hippocampal cultures produced lower release of LDH revealing decreased cell death than that observed in WT cultures (Dworzak et al., 2015). Furthermore, Aβ_1–42_ treatment impaired synaptic transmission, decreasing α-amino-3-hydroxy-5-methyl-4-isoxazolepropionic acid receptor (AMPAR)-dependent miniature excitatory postsynaptic currents (mEPSCs), suggesting both a pre- and post-synaptic compromise (Dworzak et al., 2015).

Conversely, cell loss in the purified microglia cell culture from *Cx3cr1^−/−^* mice were higher than the loss observed in WT microglia cultures. The opposite cytotoxic response induced by Aβ observed in *Cx3cr1^−/−^* microglia and neurons suggest that CX_3_CR1 endows each cell type with different effector functions.

Combining *Cx3cr1* deletion, a triple-transgenic mouse model of AD (3xTg-AD: *PS1M146V* Kin, transgenic *APPSwe* and *tauP301L*), and two-photon microscopy, it was possible to evaluate simultaneously neuronal death and microglial cell behavior during a 28-d imaging in living mice (Fuhrmann et al., 2010). A significant 2% neuronal loss in cortical layer III was observed in 4-6-month-old *3xTg-Cx3cr1^+/−^* mice. By contrast, *Cx3cr1^+/−^*, *Cx3cr1^−/−^* or 3xTg-*Cx3cr1^−/−^* mice, did not exhibit neuron loss over the same imaging period (Fuhrmann et al., 2010). Thus, *Cx3cr1* deletion appears to be neuroprotective. Additionally, in 3xTg-*Cx3cr1^+/−^* mice, microglia, showing an increased migration, were recruited to the sites of neuron loss before the death of neurons. Interestingly, microglia Aβ-phagocytosing activity was not modified by *Cx3cr1* deletion (Fuhrmann et al., 2010). This suggests that expression of CX_3_CR1 by microglia is required for neuron elimination in the context of an AD model (Fuhrmann et al., 2010), but the cellular mechanisms underlying such neuron–microglia interaction remain unknown.

Deletion of *Cx3cr1* also appears to be neuroprotective in other AD mice models. For instance, in APP/PS1 and R1.40 mice, APP/PS1 mice co-express hu APP carrying the *K670M/N671L* familial AD mutation and presenilin 1 with the *L166P* familial AD mutation leading to primarily Aβ_1-42_ oligomer aggregation. The R1.40 transgene is a full genomic copy of hu *APP* carrying the *K670M/N671L* familial AD mutation that leads to gradual deposition of Aβ_1-40_ oligomer. In 4-month-old APP/PS1 mice and in 20-24-month-old R1.40 mice, *Cx3cr^−^* deletion reduced Aβ deposition and augmented microglia accumulation around Aβ deposits. In both models, *Cx3cr1* deletion reduced immunodetection of CD68(+) microglia, whereas qRT-PCR revealed that CX_3_CR1 deficiency reduced TNFα and the chemokine (C-C motif) ligand 2 (CCL2) mRNA levels, and increased IL1β mRNA. In addition, in both AD models, *in vitro* and *in vivo* microglial phagocytosis assessment revealed enhanced Aβ uptake (Lee et al., 2010), suggestive of enhanced Aβ clearance and reduced inflammation (Lee et al., 2010).

In the brain of APP/PS1 mice heterozygous for *Cx3cr1* (APP/PS1- *Cx3cr1^+/−^*), Aβ level and senile-like plaque deposition are reduced in comparison with age-matched APP/PS1 mice. Reduced Aβ level in the brain was associated with high level of the neuronal-expressed Aβ-degrading enzymes (insulysin and matrix metalloproteinase 9) and with improved performance in the Barnes Maze cognitive test (Hickman et al., 2019). Barnes maze assesses memory and spatial learning placing a rodent on a circular arena, where, moved by its instinctive aversion to open spaces and its natural preference for dark and sheltered spaces, mice select 1 out of 20 equally distributed holes on the arena periphery, which is the unique hole equipped with the animal’s home cage.

#### 1.5.5. Parkinson’s disease

PD is the second most common neurodegenerative disease after AD, affecting about 1% of the population older than 60 years (de Lau and Breteler, 2006). PD is characterized by the gradual loss of dopaminergic neurons in the substantia nigra pars compacta (SNpc), the remaining SNpc neurons can show intracellular protein aggregates called Lewy bodies, which are mainly constituted by α-synuclein (SNCA). The cellular pathogenesis of PD is associated with mitochondrial dysfunction, oxidative stress, neuroinflammation, proteasomal dysfunction, impaired autophagy, increased protein aggregation and enhanced apoptosis (Shan et al., 2011; Angelopoulou et al., 2020). PD patients exhibit a constellation of motor and non-motor signs and symptoms. Rest tremor, rigidity, bradykinesia, and loss of postural reflexes are considered as cardinal signs (Jankovic, 2008). Motor signs also include, among others, shuffling gait with decreased arm swing, difficulty arising from chair, turning in bed, hypomimia affecting facial gestural expression and communication, dysarthria, dysphagia, micrography, glabellar reflex, blepharospasm, dystonia, and sialorrhea. Non-motor signs and symptoms include cognitive impairment, bradyphrenia, tip-of-the-tongue (word finding) phenomenon, depression, apathy, anhedonia, fatigue, other behavioral and psychiatric problems including dementia, anosmia, ageusia, pain (shoulder, back), paresthesia, dysautonomia (orthostatic hypotension, constipation, urinary and sexual dysfunction, abnormal sweating, seborrhea), weight loss, REM sleep disorders, sleep fragmentation, and restless legs syndrome (Jankovic, 2008). Current therapies provide symptomatic relief, but fail to stop disease progression (Angelopoulou et al., 2020).

Administration of 1-methyl-4-phenyl- 1,2,3,6-tetrahydropyridine (MPTP), or its active metabolite, 1-methyl-4-phenylpyridinium (MPP+), has been extensively used to model PD in non-human primates and rodents. In mice receiving IP MPTP injection, *Cx3cl1* or *Cx3cr1* deletion worsened the dopaminergic neuronal loss and increased microglial activation in the SNpc (Cardona et al., 2006).

The effects induced by IP injection of MPTP varies on different mice strains. For instance, IP injection of MPTP in C57BL/6 mice, but not into BALB/c (BALB) mice, induced behavioral dysfunction, activated microglia/astrocytes, and increase of IL10, IL12, IL13, IFNγ, and CCL2 in cerebrospinal fluid (CSF). It has been proposed that differences in the immunological background affect the outcome. B6 mice show a bias toward Th1 acquired immune responses, whereas BALB mice toward Th2, which are characterized by their distinct cytokine profiles (Yasuda et al., 2008). IL2, IFNγ and TNFα are Th1 cytokines and IL4, IL5, IL6, IL10, and IL13 is Th2 cytokines. Activation of Th1 promotes cell-mediated immunity and activation of Th2 promotes humoral immunity (Yasuda et al., 2008).

Unilateral injection of MPP+ into the rat SN increase the expression of CX_3_CL1, and CX_3_CR1 in the ipsilateral (injected) but not in the contralateral SN (Shan et al., 2011). In contrast, both proteins are weakly expressed in sham animals. On the other hand, unilateral injection of CX_3_CL1 into the rat SN increased microglial activation, dopaminergic neuron loss and motor dysfunction. After 14 days of MPP+ treatment, almost all CX_3_CR1(+) cells in the injected SN expressed CD11b, also called integrin alpha-M, ITGAM, or integrin alpha-X, ITGAX, is commonly used to identify macrophages and microglia and showed an activated microglia-like phenotype. Furthermore, daily ICV injection of anti-CX_3_CR1 neutralizing antibodies for a week after injection of MPP+ reduced, in a dose-dependent way, the MPP + -induced rotation behavior. In addition, microglia activation, dopaminergic degeneration, and CX_3_CL1-induced behavior abnormalities induced by the injection of CX_3_CL1 into the SN were prevented by ICV administration of anti-CX_3_CR1 neutralizing antibody 6 and 1 h before CX_3_CL1 injection (Shan et al., 2011). Moreover, ICV administration of minocycline, a selective microglia inhibitor, prevented CX_3_CL1-induced rotation behavior and reduced dopaminergic neuron loss in a dose-dependent way (Shan et al., 2011), supporting the involvement of microglia in the dopaminergic toxicity induced by MPP+ and CX_3_CL1.

Mice with a *Cx3cr1* deletion have reduced dopamine levels in the striatum, independently of the neurotoxin administration. After 7 days of intranasal inoculation of MPTP, mice of various genotypes (*Cx3cr1^+/+^*, *Cx3cr1^+/−^*, *Cx3cr1^−/−^*) showed a reduction in tyrosine hydroxylase (TH)- and dopamine transporter (DAT)-positive cells in the striatum. Whereas, in the SN, *Cx3cr1^+/+^* and *Cx3cr1^+/−^* genotypes showed a reduction of TH-positive cells, but in *Cx3cr1^−/−^* mice, no change of TH- or DAT-positive cells after intranasal MPTP inoculation was observed (Tristao et al., 2016).

The effects of *Cx3cr1* deletion on MPPT-treated mice do not depend only on the brain region analyzed (SN vs. striatum), but also depend on the via of MPPT administration (Tristao et al., 2016). After 3 and 7 days of intranasal MPPT, *Cx3cr1* deletion did not affect microglial activation and astrogliosis in the striatum. Although, *Cx3cr1* deletion at day 3 did not affect the loss of dopamine neurons in the striatum, it showed neuroprotective effects on day 7. In contrast, after intraperitoneal MPTP treatment, *Cx3cr1* deletion did not affect dopaminergic degeneration or astrogliosis but increased microglial activation in the striatum and SN (Tristao et al., 2016).

Another model of PD consists in the unilateral stereotaxic administration of the catecholaminergic neurotoxin 6-hydroxydopamine (6 − OHDA), in the rat striatum or SN. 6 − OHDA does not cross the BBB and induces dopaminergic degeneration essentially by increasing ROS and inflammation (Blesa et al., 2012). The administration of recombinant CX_3_CL1 via an osmotic minipump for 28 days starting 7 days after the 6-OHDA injection into the same striatum, prevented activation of microglia, and showed neuroprotective effect, reducing the loss of striatum neurons that resulted in the reduction in volume of the striatum. Application of CX_3_CL1 also reduced dopaminergic cell loss and inflammation in the SN (Pabon et al., 2011).

Transgenic PD models have been developed by overexpression of mutant genes for autosomal dominant genes such as α-synuclein and leucine rich repeat kinase 2 (LRRK2) and KO or Kdown models for autosomal recessive genes, such as Parkin, DJ-1, phosphatase and tensin homolog (PTEN)-induced novel kinase 1 (PINK1) (Dawson et al., 2010; Blesa et al., 2012; Angelopoulou et al., 2020). Increase of α-synuclein promotes neuroinflammation, astrocytosis, microgliosis, activation of microglia, and NFκB activation, leading to an increased release of inflammatory cytokines, promoting neuroinflammation and worsening dopaminergic neurodegeneration (Angelopoulou et al., 2020). Microglia activation results in an increased phagocytosis of α-synuclein, which exacerbates the production of ROS and inflammatory activation (Theodore et al., 2008; Angelopoulou et al., 2020).

CX_3_CL1 has diverse effects on neuroinflammation, and degeneration in α-synuclein models of PD (Angelopoulou et al., 2020). CX_3_CL1 can be neuroprotective, inhibiting motor impairment and dopaminergic cell loss in the SN and striatum of rats injected with rAAVs coding for overexpressed hu α-synuclein (Nash et al., 2015). Overexpression of hu α-synuclein or hu α-synuclein A53T in WT (*Cx3cr1^+/+^*) or null (*Cx3cr1^−/−^*) mice display very low levels of CX_3_CL1. Interestingly, overexpression of hu α-synuclein induced microgliosis, neuroinflammation, and dopaminergic neuronal death in SN, which are of similar magnitude in *Cx3cr1^+/+^* or *Cx3cr1^−/−^* mice. However, the overexpression of hu α-synuclein A53T resulted in an exacerbated neurodegeneration compared to hu α-synuclein overexpression. Moreover, α-synuclein A53T-induced neurodegeneration was enhanced in *Cx3cr1^−/−^* mice (Castro-Sanchez et al., 2018).

### 1.6. CX_3_CL1/CX_3_CR1 axis and aging

CX_3_CL1 expression is reduced in the brain of aged rats, being associated to an age-related increase of microglial cell activation (Lyons et al., 2009). Levels of sCX_3_CL1 decrease with aging, which could lead to an enhanced inflammation, deficits in synaptic remodeling, and eventually to cognitive impairment (Winter et al., 2020). Consistent with this, treatment of aged rats with CX_3_CL1 blunt the age-related increase in microglial activation (Lyons et al., 2009). Aged (18–22 m) BALB/c mice show a reduced level of basal total CX_3_CL1 in cortex and hippocampus and the level of CX_3_CL1 is unchanged 24 h after LPS IP injection, compared with the response observed in young (3–6 m) mice (Wynne et al., 2010). CX_3_CR1 level in old mice is also lower than in young ones.

#### 1.6.1. CX_3_CL1 cleavage and aging

The native 95 kDa membrane-bound CX_3_CL1 (mCX_3_CL1) can be transformed into a 65 kDa chemotactic glycoprotein (sCX_3_CL1, which contains the extracellular N-terminal CKD and MS domains), by metalloproteinases such as the TNFα converting enzyme (TACE, ADAM17) (Garton et al., 2001; Tsou et al., 2001), ADAM10 (α-secretase) (Hundhausen et al., 2003, 2007), matrix metalloproteinase 2 (MMP-2) (Bourd-Boittin et al., 2009) or the Cathepsin S (CatS) produced by microglia (Clark et al., 2009). CX_3_CL1 is also a substrate for β-secretase (BACE1), and γ-secretase, involved in the APP cleavage to generate Aβ (Fan et al., 2019).

Age-dependent changes in CX_3_CL1 shedding can be the result of changes in the amount of protease available to process mCX_3_CL1, as consequence of changes in their synthesis or degradation. Alternatively, age-dependent changes in the enzymatic efficiency of proteases may be caused by changes in the amount of endogenous inhibitors or enzyme enhancers (Postina, 2012; Mathews and Levy, 2016; Shi et al., 2018; Sharma et al., 2022).

ADAM10 and ADAM17 are the best characterized members of the disintegrin and metalloprotease (ADAM) family (Black and White, 1998) involved in the processing of membrane-associated proteins such cytokine receptors, chemokines, adhesion molecules, and growth factors. Their targets include TNFα, TGFβ, Notch, CX_3_CL1, APP, the low-density lipoprotein receptor-related protein 1 (LRP1), and the anti-aging protein α-klotho (Blobel, 1997; Peschon et al., 1998; Borrell-Pages et al., 2003; Liu et al., 2009; Chen et al., 2014; Brifault et al., 2017).

The enzymes, ADAM 10, and ADAM 17 are implicated in the α-secretase non-amyloidogenic APP processing, leading to the production of soluble APP (APPsα), which exhibits properties of neuroprotection, memory-enhancer, and regulation of neuronal excitability, synaptogenesis, and synaptic plasticity (Turner et al., 2003; Reinhard et al., 2005; Zheng and Koo, 2006). Mass spectrometric analysis revealed that 23-month-old rats showed a reduction of soluble APPsα in cisternal CSF compared with that observed in 3- and 13-month-old animals (Anderson et al., 1999). The reduction of APPsα has a positive correlation (r = 0.52–0.57, *p* < 0.001) with the performance in spatial memory tasks observed in young and aged rats (Anderson et al., 1999). Accordingly, ADAM 17 activity in cortex and hippocampus changes with aging (Bertoldi et al., 2017). Aged rats show a reduced ADAM 17 activity, whereas the amyloidogenic BACE activity is like that in young rats (Bertoldi et al., 2017). This age-dependent reduction in ADAM 17 activity is accompanied with a reduced performance in aversive memory test (Bertoldi et al., 2017). By contrast, daily moderate treadmill exercise for two weeks had not impact on ADAM 17 and BACE activities at various ages (Bertoldi et al., 2017).

CatS is a member of the family of cysteine lysosomal proteases preferentially expressed in macrophages and microglia and released from them in response to neurotrophic factors and inflammatory mediators. It plays a role, together with ADAM 17 and ADAM10, in CX_3_CL1 shedding (Petanceska et al., 1996; Liuzzo et al., 1999a,b; Turk et al., 2000; Nakanishi, 2003; Wendt et al., 2008).

Age-dependent changes in the protein expression of CatS has been detected by Western Blot in cerebral cortex, cerebellum, brainstem, and spinal cord. In 1-wk-old mice, only pro- CatS is found. Starting at 6 months, age-dependent upregulation of prepro-form, pro-form, and mature form of CatS is evidenced (Wendt et al., 2008). Furthermore, analysis of gene expression by DNA microarrays reveals that CatS gene expression was higher in 25-month-old mice than that observed in 5-month-old mice (Park et al., 2009).

Double immunofluorescent labeling revealed that most CatS positive cells in the CNS are microglia (PT66), followed by astrocytes (GFAP) and a few neurons (Neu) or endothelial cells (von Willebrand factor). Aging upregulates the number and the labeling intensity of CatS positive cells. The number of glia-like cells as well as neurons expressing CatS in cortex and brainstem clearly increases in the aged mouse brain (Wendt et al., 2008).

Age-dependent changes in the activity of CatS are not only due to the increased, but also to age-dependent changes of the level of the cysteine-protease inhibitor cystatin C (CysC, also known as γ-trace), an endogenous inhibitor of CatS, a member of the endogenous cysteine-protease inhibitors (Barrett et al., 1986; Mathews and Levy, 2016), which is found in neurons, astrocytes, endothelial, and microglia cells (Yasuhara et al., 1993; Palm et al., 1995; Miyake et al., 1996). Besides of inhibiting CatS, CysC also inhibits the cysteine-proteases cathepsin (Cat) B, Cat H, Cat K, and Cat L (Turk et al., 2008). In turn, CysC is targeted and inactivated by aspartyl-protease Cat D and the serine-protease elastase (Lenarcic et al., 1991; Abrahamson et al., 1991a,b).

Hu CysC is a 120 aa protein which is preceded by a26 aa amino terminal that endows it with the capacity of being secreted (Turk and Bode, 1991; Turk et al., 1997). The novo synthesized CysC can access the endosomal-lysosomal compartment via an intracellular pathway or most of it is secreted to the extracellular compartment (Zucker-Franklin et al., 1987; Chapman et al., 1990; Barka et al., 1992; Tavera et al., 1992; Paraoan et al., 2001) and, only a fraction remains within exosomes (Ghidoni et al., 2011). Secreted CysC can be found in cell surfaces or in the extracellular matrix (Calkins and Sloane, 1995; Sastre et al., 2004; Kolodziejczyk et al., 2010), where it can inhibit the activity of released cathepsins, or alternatively, it can be internalized into other cells (Merz et al., 1997; Ekstrom et al., 2008). CysC is internalized via endocytosis, allowing its access into the endosomal-lysosomal compartment (Merz et al., 1997; Pierre and Mellman, 1998; Ekstrom et al., 2008) where it can inhibit lysosomal cathepsins and be degraded by aspartyl-protease Cat D (Wallin et al., 2013).

CysC secretion appears to be age-dependent since CysC levels in serum correlates with age in dogs, cats, and humans (Ohara et al., 2012; Ghys et al., 2015; Iwasa et al., 2022). Changes of CysC brain expression and secretion are found in several neurological disorders, and also correlates with animal models of neurodegeneration (Mathews and Levy, 2016). However, up to date, conclusive studies on age-dependent changes in CysC expression are lacking.

### 1.7. Differential effects of CX_3_CL1 forms

**Figure 3:**
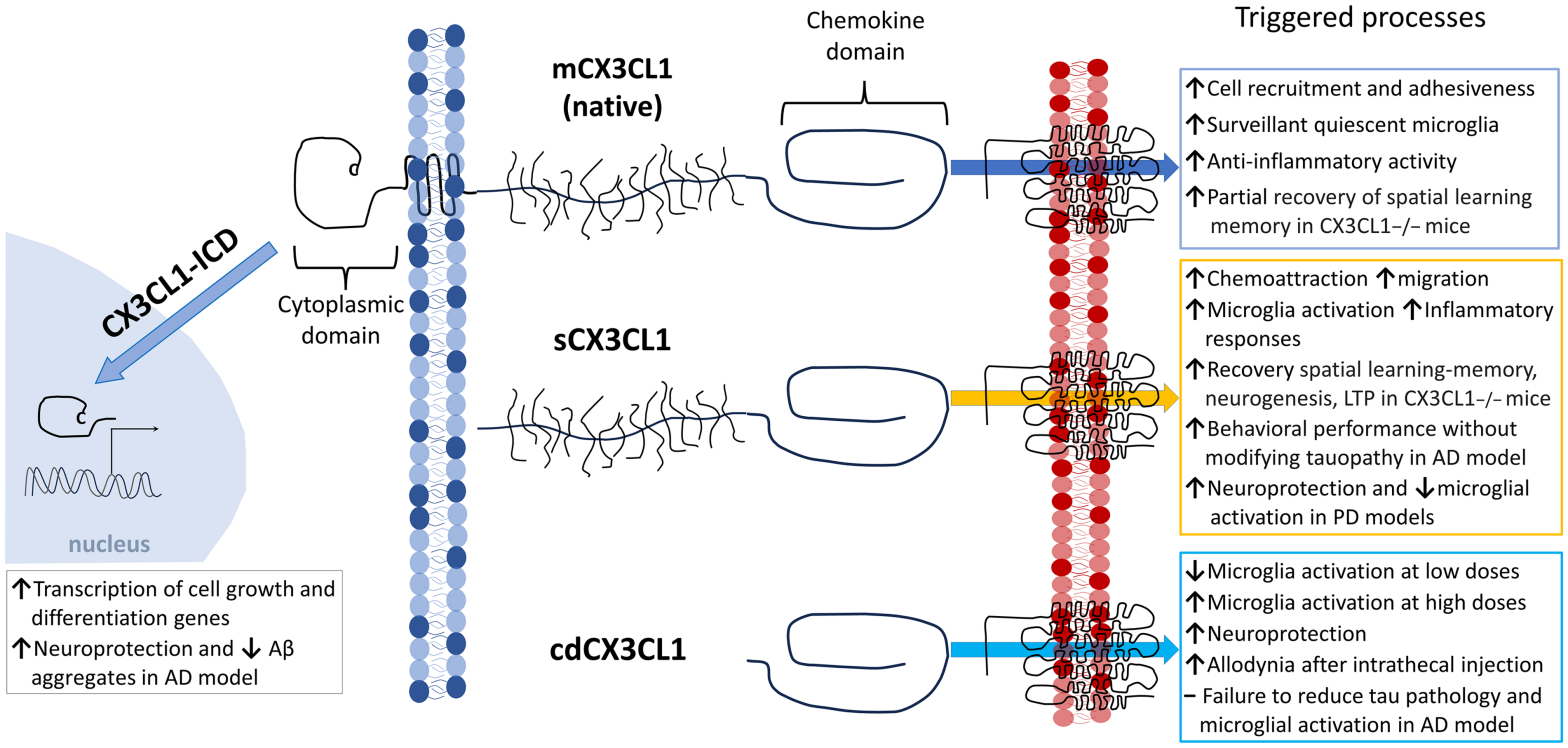
Differential functions of the various Fractalkine forms. Scheme illustrating different processes triggered by the activation of microglia by CX_3_CR1 binding mCX_3_CL1, sCX_3_CL1, and cdCX_3_CL1. Newly reported CX_3_CL1-ICD proposed functions are included. Within sCX3CL1s, we considered fragments resulting from ectodomain shedding or recombinant synthesis based on the action of proteases. Note that cdCX_3_CL1 is considered to be independent from sCX_3_CL1.

As described, sCX_3_CL1 with different sizes are the result of cleavage of the mCX_3_CL1 by proteases (Bazan et al., 1997; Figures 1, 3). Notoriously, both variants showed selective functions (Bazan et al., 1997). sCX_3_CL1 have a potent chemoattractant activity for T cells and monocytes, favoring cellular migration and recruitment, whereas the mCX_3_CL1 endowed primary endothelial cells with recruitment and strong adhesion to leukocytes (Bazan et al., 1997; Imai et al., 1997; Hermand et al., 2008; Ostuni et al., 2014). However, mCX_3_CL1 and sCX_3_CL1 variants can also share functions, for instance, they can reduce the expression of microglia pro-inflammatory genes, like NOS, IL1β, TNFα, and IL6, attenuate LPS-induced microglial cell activation *in vitro*, the age-related increase in microglial cell activation, and reduce neuronal death induced by microglia activated by IFNγ or LPS (Zujovic et al., 2000; Mizuno et al., 2003; Lyons et al., 2009).

What is striking are the findings that binding of mCX_3_CL1 with CX_3_CR1 acts as a cell–cell adhesion molecule and has anti-inflammatory activity, keeping the microglia in a state of surveillance (Ludwig and Mentlein, 2008; Lee et al., 2014; Williams et al., 2014; Mecca et al., 2018; Figure 3). By contrast, the binding of sCX_3_CL1 with CX_3_CR1 can induce inflammatory activation of microglia, having a proinflammatory and a chemoattractant effect for microglia and other innate immune cells (Jones et al., 2010). Differential functions between mCX_3_CL1 and sCX_3_CL1 were observed in the APP/PS1 mouse model, in which *Cx3cl1*-deficient APP/PS1 animals exhibited reduced Aβ deposition but enhanced neuronal tau phosphorylation (Lee et al., 2014). Surprisingly, reduced Aβ deposition and enhanced neuronal tau phosphorylation were unmodified by the transgenic expression of sCX_3_CL1 isoforms, suggesting that mCX_3_CL1 regulates both processes (Lee et al., 2014).

Physiologically, the CX_3_CL1/CX_3_CR1 axis appears to be involved in the regulation of cognition and memory (Rogers et al., 2011). For instance, it has been reported that sCX_3_CL1 is beneficial in rescuing memory, whereas the mCX_3_CL1 lacks this effect (Winter et al., 2020). *Cx3cl1^−/−^* mice exhibit a significant cognitive impairment in a fear conditioning task for long-term memory and in the Barnes maze task for assessing spatial learning and memory (Winter et al., 2020). These deficits correlate with reduced hippocampal neurogenesis and LTP. After 2 months of the injection of rAAV expressing a mutated, obligate membrane-bound form of mCX_3_CL1 or a sCX_3_CL1 in *Cx3cl1^−/−^* mice, CX_3_CL1 was detected by ELISA in brain tissue. In *Cx3cl1^−/−^* mice injected with rAAV expressing mCX_3_CL1, CX_3_CL1 level was like that of WT mice, whereas in mice injected with AAV expressing sCX_3_CL1, high levels of CX_3_CL1 (almost doubling WT level) were observed. The *Cx3cl1^−/−^* mice treated with rAAV expressing mCX_3_CL1 showed an improved performance in the accelerating rotarod test compared to WT mice, whereas those injected with rAAV expressing sCX_3_CL1, showed similar motor performance to WT. Remarkable, treatment with AAV-mCX_3_CL1 partially restored spatial learning and memory in *Cx3cl1^−/−^* mice, but did not rescue long-term memory, or neurogenesis (Winter et al., 2020). In contrast, treatment with AAV-sCX_3_CL1 partially corrected changes in both cognitive and motor function and restored neurogenesis and LTP to levels like WT animals (Winter et al., 2020). These results could indicate that treatments based on restoration of the level of sCX_3_CL1 may be a viable therapeutic target for dysfunctions in aging and disease. How much of these differences rely on the CX_3_CL1 levels attained by each kind of transgenic or in the functional capacity of mutated mCX_3_CL1 protein in the experiments (Winter et al., 2020) remain as open questions.

To evaluate the impact of sCX_3_CL1 forms upon pathological hallmarks of AD, Lee (Lee et al., 2014) used a mouse line expressing exclusively sCX_3_CL1, obtained by introducing an artificial chromosome transgene encoding truncated CX_3_CL1 (SolTg) to *Cx3cl1^−/−^* mice. These *Cx3cl1^−/−^*; SolTg and *Cx3cl1^−/−^*mice were mated with APP/PS1 mice, resulting in APP/PS1; *Cx3cl1^−/−^*; SolTg and APP/PS1; *Cx3cl1^−/−^* mouse lines, respectively. As expected, CX_3_CL1-deficient APP/PS1 mice, as previously reported for CX_3_CR1-deficient APP/PS1 mice (Lee et al., 2010), also exhibited reduced Aβ deposition on cortex and hippocampus compared with APP/PS1 mice. However, APP/PS1;*Cx3cl1^−/−^*;SolTg mice brains also exhibited reduced Aβ deposition with levels undistinguishable from those in *Cx3cl1^−/−^*- and *Cx3cl1^−/−^*- deficient APP/PS1. Surprisingly, despite reduced Aβ deposition, *Cx3cl1^−/−^*-deficient APP/PS1 mice demonstrated elevated phosphorylated tau levels (Lee et al., 2014). In fact, Lee et al. (2014) proposed that the absence of mCX_3_CL1 reduces Aβ deposition via p38 MAPK-mediated activation of microglia and the SRA (macrophage scavenger receptor 1), (encoded by *msr1*)-dependent phagocytosis (Lee et al., 2014; Cornejo et al., 2018). Nevertheless, the assignation of mCX_3_CL1 functions by subtraction or default should be taken with caution, because no direct manipulation of mCX_3_CL1 was performed and a measurement of the level of sCX_3_CL1 in the APP/PS1 *Cx3cl1^−/−^*;SolTg mice were not performed. If the amount, regulation, or activity of metalloproteases are different in APP/PS1 *Cx3cl1^−/−^*;SolTg mice, the level of CX_3_CL1 active fragments could change.

Disruption of CX_3_CL1 signaling has shown to have opposite effects on Aβ and tau aggregation on mouse AD models. The sCX_3_CL1 overexpression in the hippocampus does not affect Aβ deposits and microglia activation found in the rTg4510 mouse model (Nash et al., 2013). Conversely, as described in the section of neurodegenerative diseases, Nash et al. (2013) show that the restricted focalized overexpression of sCX_3_CL1 in the hippocampus of the rTg4510 mouse model can ameliorate tauopathy, without significant improvement in performance in the radial arm water maze (Nash et al., 2013). Notably, through a change in the methodological strategy, 5-month-old rTg4510 mice received ICV injections with serotype 4 AAV (AAV4) coding for sCX_3_CL1. This AAV4 has the capacity of easily infect cells lining the ventricular system in the CNS. Thus, sCX_3_CL1 was overexpressed by cells lining the ventricles, which was associated with significantly improved cognitive performance on the mouse recognition and radial arm water maze (Finneran et al., 2019). Behavioral benefits were attained without reduction in tau hyperphosphorylation, hippocampal atrophy, or microglial cell number (Finneran et al., 2019).

Recently, it was reported that recombinant full-length hu sCX_3_CL1 and the small soluble fragment constituted by its chemokine domain (cdCX_3_CL1) show, depending on the assay, functional differences (Finneran et al., 2023). Both CX_3_CL1 isoforms show modest differences in their receptor affinity but similar potency and efficacy using a cell-based assay of CX3CR1-dependent reduction in forskolin-induced cAMP.

By contrast, sCX_3_CL1 show a 10-fold higher potency than that of cdCX3CL1 in the β-arrestin recruitment assay. Furthermore, sCX_3_CL1 is also 10-fold more potent than cdCX_3_CL1 in decreasing LPS-induced TNFα release by microglia, which suggests that CX_3_CR1 signaling through β-arrestin may be involved in the anti-inflammatory effect of CX_3_CL1. When recombinant sCX_3_CL1 is administered at concentrations above normal basal plasma level, it induces an increase in the inflammatory microglial activation. The opposite effect is observed with cdCX_3_CL1, but it is 10-fold less potent (Finneran et al., 2023).

Differential effects elicited by sCX_3_CL1 and cdCX_3_CL1 has also been reported on neuropathic pain. In response to nerve injury, microglial activation and CX_3_CL1 expression are increased. CX_3_CR1 antagonists can relieve nerve injury-induced pain in rats (Milligan et al., 2004; Sun et al., 2013; Sessler et al., 2021). Furthermore, intrathecal administration of cdCX_3_CL1 but not sCX_3_CL1 enhance mechanical allodynia, whereas inhibition of CatS has antihyperalgesic and antiallodynic effects in a neuropathic pain rat models and reduced microglia activation in the spinal cord (Clark et al., 2007). In fact, intrathecal administration in the spinal cord of recombinant CatS induce hyperalgesia and allodynia in naïve rats (Clark et al., 2007). CatS generates a 55 KDa sCX3CL1 (Fonovic et al., 2013) shorter than that generated by ADAM10/17 (about 85 KDa) (Roche et al., 2016) and similar in size to the 76 aa of the chemokine domain.

To evaluate the effect of cdCX_3_CL1 on tau pathology and its behavioral impact, a mouse line (CX_3_CL1^105Δ^) was developed. CX_3_CL1^105Δ^ expresses exclusively cdCX_3_CL1, lacking the mucin stalk, and overexpressing hu Tau protein for developing tau neurofibrillary pathology (Bemiller et al., 2018). Despite of being overexpressed, cdCX_3_CL1 fails to reduce tau pathology and microglial activation (Bemiller et al., 2018). In addition, expression of CX_3_CR1 in the microglial cell membrane is significantly reduced in CX_3_CL1^105Δ^ mice, mimicking a CX_3_CR1 deficiency, suggesting that cdCX_3_CL1 overexpression downregulates CX_3_CR1 expression by microglia and increases tau pathology. Similar results were observed using other AD mice models carrying the APP/PS1 double mutation and expressing only the chemokine domain of CX_3_CL1 (CX_3_CL1^105Δ^). APP/PS1:CX_3_CL1^105Δ^ mice presented a more severe pathological phenotype and time course than that observed in APP/PS1 mice lacking the entire gene (APP/PS1:*Cx3cl1^−/−^*) (Lee et al., 2014).

Additionally, recent reports show that successive cleavage of mCX_3_CL1 by β- and γ-secretases, release the intracellular domain of CX_3_CL1 (CX_3_CL1-ICD), which translocate to the cell nucleus and regulates the transcription of several genes important for cell growth and differentiation (Fan et al., 2019). Interestingly, Tg-CX_3_CL1-ICD mice did not exhibit overgrowth, whereas 5xFAD, a mouse model of AD that overexpresses CX_3_CL1-ICD exhibits reduced Aβ deposition and neuronal loss, the latter, likely due, to enhanced neurogenesis. These results demonstrate a beneficial effect of the CX_3_CL1-ICD in an AD mouse model independent of the activation of CX_3_CR1 (Fan et al., 2019).

The role of soluble and membrane-bound CX_3_CL1 has also been examined in PD mouse models (Morganti et al., 2012; Nash et al., 2015). SNpc injections of AAV vectors for sCX_3_CL1, a mutant mCX_3_CL1 (with mutations R337A + R338A that abolish ADAM10/17 cleavage into sCX_3_CL1) or a control vector expressing GFP were performed in 3-4-month-old *Cx3cl1^−/−^* mice. Six weeks after the viral vector injection, mice received an intraperitoneal MPTP injection to induce PD-like disorder (Morganti et al., 2012). The soluble, but not the mCX_3_CL1 reduced the MPTP-induced motor impairment, dopaminergic neurons loss, and microglial activation in the SNpc. These results were associated to the decreased release of inflammatory cytokines (TNFα and IL1β) and microglial expression of CD68 and CD11b (Morganti et al., 2012). These results suggest that in PD, the ADAM10/17 cleavage variant sCX_3_CL1 (aa 1–336) appears to be the one that confers neuroprotection and not the mCX_3_CL1.

The role of various molecular forms of CX_3_CL1 was also studied in a rat model of PD induced by SNpc injection of rAAV coding for hu α-synuclein in rats (Nash et al., 2015). Rats that received a co-injection of viral vectors coding for hu α-synuclein and sCX_3_CL1 exhibited reduced loss of tyrosine hydroxylase and Neu-N labeling in the SNpc, indicative of neuroprotection of dopaminergic neurons. Co-injection of viral vectors carrying other fractalkine forms showed a loss of dopaminergic neurons comparable to those observed in controls receiving injection of huα-synuclein alone or coinfected with the control viral vector coding for GFP. Similar neuroprotective effect was observed in animals expressing fractalkine forms in astrocytes, thanks to AAV constructs containing the fibrillary acid protein (GFAP) promoter (Nash et al., 2015).

## 2. Conclusion

Microglia are essential components of the homeostatic response of the CNS. They are constantly under the influence of “off” and “on” signals involved in the dialog between microglia and other CNS cells, both in physiological and pathological settings. ‘Off signals” tend to maintain microglia in a surveillant status and include the CX_3_CL1/CX_3_CR1 and CD200/CD200R axes, with ligands presented by neurons and their receptors by microglia, soluble mediators like TGFβ, IL10, IL34, and colony stimulating factor 1 (CSF1), which are secreted by healthy neurons and their receptors, TGFβR and CSF1R, are present, among many cells, in microglia, as well as receptors for different neurotransmitters (glutamate, GABA, acetylcholine and noradrenaline) (Manich et al., 2019). Thus, in various models of neuroinflammation and neurodegeneration, impairment of the CX_3_CL1/CX_3_CR1 or CD200/CD200R axes, or similarly, when microglial receptors for TGFβ or CSF1 are deleted, microglia activation become more neurotoxic (Hoek et al., 2000; Cardona et al., 2006; Buttgereit et al., 2016). On the other hand, “on” signals that activate microglia, include soluble factors like cytokines/chemokines, trophic factors, having receptors on activated microglia. In addition, microglia present several membrane-bound receptors that recognize and bind molecules expressed by damaged neurons (Manich et al., 2019). Thus, a constellation of molecules regulates microglial function through numerous molecules. The fact that fractalkine axis is only one among multiple molecular systems contributing to the regulation of microglia, should be a warning note for interpreting negative results obtained through the manipulation of a single component of the CX_3_CL1/CX_3_CR1 axis. It is possible that homeostatic changes in other system could counterbalance the effects of CX_3_CL1 or CX_3_CR1 deletion.

Current evidence stress the importance of the various forms of CX_3_CL1 exhibiting regulatory roles on several physiological processes that include neuronal cell migration, synaptic pruning, synaptic maturation, microglial activation, neuroprotection, and cognitive functions. Dual pro- and anti-inflammatory effects relying on different CX_3_CL1 forms may represent a strategy for achieving brain homeostasis under various environmental conditions. Therefore, the regulation of both the level of CX_3_CL1 and the balance among its various forms, appears as a fine-tuning leading to the regulation of microglial functional state and the release of pro- or anti-inflammatory cytokines in response to the microenvironment challenges.

Thus, the search for identifying functional forms of CX_3_CL1 has yield several forms that could be separated in two categories, mCX_3_CL1 anchored to the neural plasma membrane and sCX_3_CL1, resulting from the cleavage of mCX_3_CL1 by metalloproteinases ADAM17, ADAM10 (α-secretase), CatS, matrix metalloproteinase 2 (MMP-2), or secretases involved in the Aβ processing. Each protease helps to generate a different sCX_3_CL1 form with a different size and apparently some functional properties, in addition to some newly found additions like the intracellular regions or the chemokine domain, which still require additional scrutiny.

Most of sCX_3_CL1 and mCX_3_CL1forms have in common the chemokine domain. However, the human full-length recombinant sCX_3_CL1 is 10-fold more potent in biological assays, in reducing LPS-mediated TNFα release, and for increasing inflammatory activation of microglia than cdCX_3_CL1 (Finneran et al., 2023). On the other hand, intrathecal administration of cdCX_3_CL1 but not sCX_3_CL1 enhanced mechanical allodynia and spinal cord microglia activation (Clark et al., 2007). The main differences in potency can be explained by conformational changes in the chemokine domain when it is separated from the mucine-like stalk domain, whereas the major allodynia may be the consequence of a better access of smaller proteins into the spinal cord structures. Nevertheless, up to now, it is not understood how different CX_3_CL1 forms acting on a sole CX_3_CR1 could trigger different, even opposite cellular responses, yet. Why the mCX_3_CL1 has, in general, an anti-inflammatory effect, whereas binding to the sCX_3_CL1 results in an inflammatory deleterious activation? A possible explanation to this question may be related to the fact that sCX_3_CL1 lose the property of physical anchoring between neurons and microglia, property that is only observed for the mCX_3_CL1. Such intimate cell-to-cell interaction could promote neuron–glia interactions required by other trophic factors and membrane-bound proteins like CD200 (Zhang et al., 2018) facilitating the activation of parallel intracellular pathways. To answer this puzzling and central question will allow us to understand the regulatory role of CX_3_CL1 in the cytotoxic activation leading to neurodegeneration.

Recently, the rule that all CX_3_CL1 forms perform their functions by binding CX_3_CR1 has been challenged by at least one exception, the intracellular domain of fractalkine, CX_3_CL1-ICD. CX_3_CL1-ICD is generated through the successive cleavage of mCX_3_CL1 by β- and γ-secretases. After being released in the cytoplasm, it is translocated into the cell nucleus for regulating genes important for cell growth and differentiation. This means that the fractalkine system has a wider spectrum of functions than originally thought.

### 2.1. Outlook

Worth emphasizing, the CX_3_CL1/CX_3_CR1 axis is under the influence of regulatory factors affected by the functional status of the brain, being modified by aging and inflammatory activation. Aging is associated with neuroinflammation and is the main risk factor for most neurodegenerative diseases. It has been established that the level expression of CX_3_CL1 and CX_3_CR1, the level of proteases and their inhibitors, and the microglial reactivity are all affected by aging. More studies are needed to improve our comprehension of aging effects and their impact on the activity ratios among different forms of CX_3_CL1.

The potential therapeutic role of the CX_3_CL1/CX_3_CR1 axis has been explored in animal models with controversial results. Further study requires a more complete and extensive description of the multiple factors associated with the experimental manipulation. The lack of an exhaustive evaluation of the functional properties of genetic constructs could mask an artefactual loss of affinity or steric impairment for an efficient binding, among several additional considerations that should be considered.

## Author contributions

JE conducted literature search and wrote the manuscript. LE-vB drew the figures and conducted literature search. RB conducted literature search, reviewed, and edited the manuscript. All authors contributed to the article and approved the submitted version.

## Funding

The current study was supported by grants Fondo Nacional del Desarrollo de la Ciencia y Tecnología (FONDECYT) 1211359 (JE) y 1221028 (RB); ANID Redes ‑190187 (RB).

## Conflict of interest

The authors declare that the research was conducted in the absence of any commercial or financial relationships that could be construed as a potential conflict of interest.

## Publisher’s note

All claims expressed in this article are solely those of the authors and do not necessarily represent those of their affiliated organizations, or those of the publisher, the editors and the reviewers. Any product that may be evaluated in this article, or claim that may be made by its manufacturer, is not guaranteed or endorsed by the publisher.
